# Mas Signaling Potentiates Neutrophil Extracellular Traps Formation Induced by Endothelial Cells Derived S1P in Mice with Acute Liver Failure

**DOI:** 10.1002/advs.202411428

**Published:** 2025-04-26

**Authors:** Bo Yang, Shuai Chen, Xiaoqi Xia, Ziwen Tao, Chun Liu, Shanshan Li, Shuo Zhang, Jiali Huang, Lu Xia, Wenqiang Quan, Changqing Yang, Jing Li

**Affiliations:** ^1^ Department of Gastroenterology and Hepatology Tongji Hospital School of Medicine Tongji University Shanghai 200065 China; ^2^ Department of Laboratory Medicine Tongji Hospital School of Medicine Tongji University Shanghai 200065 China

**Keywords:** Mas, sphingosine 1‐phosphate, neutrophil extracellular traps, farnesoid X receptor, endothelial cells

## Abstract

Mas, a newly identified G‐protein‐coupled receptor, is prevalent in myeloid‐derived immune cells and plays a key role in inflammation. This study investigates Mas signaling and neutrophil extracellular traps (NETs) in acute liver failure (ALF), aiming to elucidate their mechanisms. Male *Mas1* ^−/−^ and wild‐type mice, aged 6–8 weeks, receive intraperitoneally injected with lipopolysaccharide (LPS)/D‐galactosamine (D‐Gal) (L/G) to study NETs formation. Hepatic Mas expression increases in WT‐L/G mice, whereas systemic *Mas1* knockout significantly reduces L/G‐induced NETs and hepatotoxicity. Antibiotics treatment and co‐housing (*Mas1* ^−/−^‐L/G and WT‐L/G mice) experiments show that gut flora influences the disease phenotype in *Mas1* ^−/−^‐L/G mice. Fecal metabolite analysis suggests that mice may be protected by reduced deoxycholic acid (DCA) production in *Mas1* ^−/−^ activated hepatic farnesoid X receptor (FXR), suppressing sphingosine‐1‐phosphate (S1P)‐dependent NETs. Additionally, *Mas1* ^−/−^ also activates the FXR‐S1P‐NETs axis in the liver by inhibiting SHP2. Single‐cell sequencing shows decreased interaction between endothelial cells and Cldn1^+^CD177^+^ senescent neutrophils through Col4a1‐CD44. This inhibits S1P‐induced Raf signaling pathway activation and NETs formation. Mas signaling significantly impacts NETs formation, highlighting its potential as an anti‐inflammatory therapeutic target for ALF.

## Graphical abstract

In mice challenged with lipopolysaccharide/D‐galactosamine (L/G), Mas signaling increased SHP2 in hepatic Col4a1^+^ endothelial cells, inhibiting farnesoid X receptor (FXR), potentially due to enterogenic deoxycholic acid. This inhibition activates sphingosine‐1‐phosphate‐induced Raf/MEK/ERK signaling in Cldn1^+^CD177^+^ neutrophils, causing neutrophil extracellular traps formation and worsening liver inflammation. Moreover, Mas signaling enhances endothelial‐neutrophil interactions via Col4a1‐Cd44.

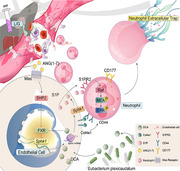

## 1 Introduction

Acute liver failure (ALF) is a critical hepatic insult characterized by a rapid onset and various etiologies, with systemic inflammatory response syndrome (SIRS) serving as a defining feature.^[^ ^1^, ^2^ ^]^ This pathological condition impairs the essential functions of the liver and has a high short‐term mortality rate reaching up to 30%.^[^ ^3^ ^]^ The incidence of ALF has significantly increased in recent years.^[^ ^4^ ^]^ The etiology of ALF varies globally. In developing nations, hepatotropic and non‐hepatotropic viruses are primary causes of ALF, whereas drug toxicity is the leading cause in Western countries.^[^ ^5^ ^]^ Despite the substantial ALF burden, however, the therapeutic options remain limited. Liver transplantation is the only proven method of extending patient survival. Therefore, understanding the pathogenesis of ALF has profound clinical significance.

Recent studies have re‐evaluated the role of the renin‐angiotensin system (RAS) in inflammation.^[^ ^6^ ^]^ Mas, a G protein‐coupled receptor encoded by the *Mas1* oncogene, is widely expressed in both human and murine tissues, and exhibits particularly high levels in myeloid immune cells. Its endogenous ligand, angiotensin (ANG)‐(1‐7), is part of the “regulatory” RAS and is recognized for its anti‐inflammatory properties and its role in mitigating tissue damage.^[^ ^7^, ^8^, ^9^ ^]^ In recent years, our research group focused on elucidating the role of Mas in acetaminophen‐induced acute liver injury (AILI). We demonstrated the protective effects of activated Mas receptor, derived from multiple cellular sources against AILI. Specifically, hepatocyte‐Mas activation enhances lipophagy and fatty acid oxidation, while myeloid‐Mas activation suppresses glycolysis to mitigate inflammation.^[^ ^10^, ^11^ ^]^ However, two renal studies have shown conflicting effects of tubular Mas activation: exacerbating lipid‐induced cell injury through autophagy and endoplasmic reticulum stress, and amplifying inflammation via NF‐κB activation in mice with ischemia/reperfusion injury.^[^ ^12^, ^13^ ^]^ Therefore, further investigation is warranted to determine the precise role of Mas in ALF, as its pleiotropic effects appear to depend on the tissue/cell type and disease context.

The liver can regenerate under mild stress; however, excessive stress can result in severe inflammation and hepatocyte death. In ALF, immune cells are recruited to the liver by inflammatory mediators, a process exacerbated by damage associated with molecular patterns (DAMPs). This cascade results in extensive immune‐mediated hepatocyte necrosis, referred to as secondary liver injury.^[^ ^14^ ^]^ Lipopolysaccharide (LPS) in combination with D‐galactosamine (D‐Gal) (L/G) is frequently employed to induce endotoxemia and selectively target hepatic tissue, thereby facilitating the development of ALF mouse models that closely mimic liver failure associated with human hepatitis B virus (HBV) infection. In recent years, sphingolipids have gained increasing attention in the context of inflammatory responses owing to their dual recognition as integral components of the cell membrane and as bioactive molecules with diverse effects.^[^ ^15^, ^16^ ^]^ To date, little is known about the role of sphingolipids in ALF. Sphingosine‐1‐phosphate (S1P), a critical sphingolipid that regulates endothelial integrity and the immune response, is significantly correlated with the severity of acute‐on‐chronic liver failure (ACLF).^[^ ^17^ ^]^ A recent study on ischemia‐reperfusion injury showed that S1P can activate S1P receptors on neutrophils, leading to the formation of neutrophil extracellular traps (NETs), which are characterized by DNA ejection and inflammatory trapping.^[^ ^16^ ^]^ The previous studies on liver injury, including ours, have highlighted the pivotal role of Mas receptor in regulating fatty acids and glycolipids.^[^ ^10^, ^18^ ^]^ However, its association with the sphingolipid metabolism remains unclear.

This study conducted a series of in vivo and in vitro experiments utilizing the *Mas1* ^−/−^‐L/G mouse model to assess the regulation of S1P‐dependent NETs by Mas in the progression of ALF. The findings offer novel insights into the development of pharmacological interventions for the management of ALF.

## 2 Results

### 2.1 Intrahepatic Mas Receptor Expression is Significantly Upregulated in ALF Patients and Mouse Models

**Figure 1:**
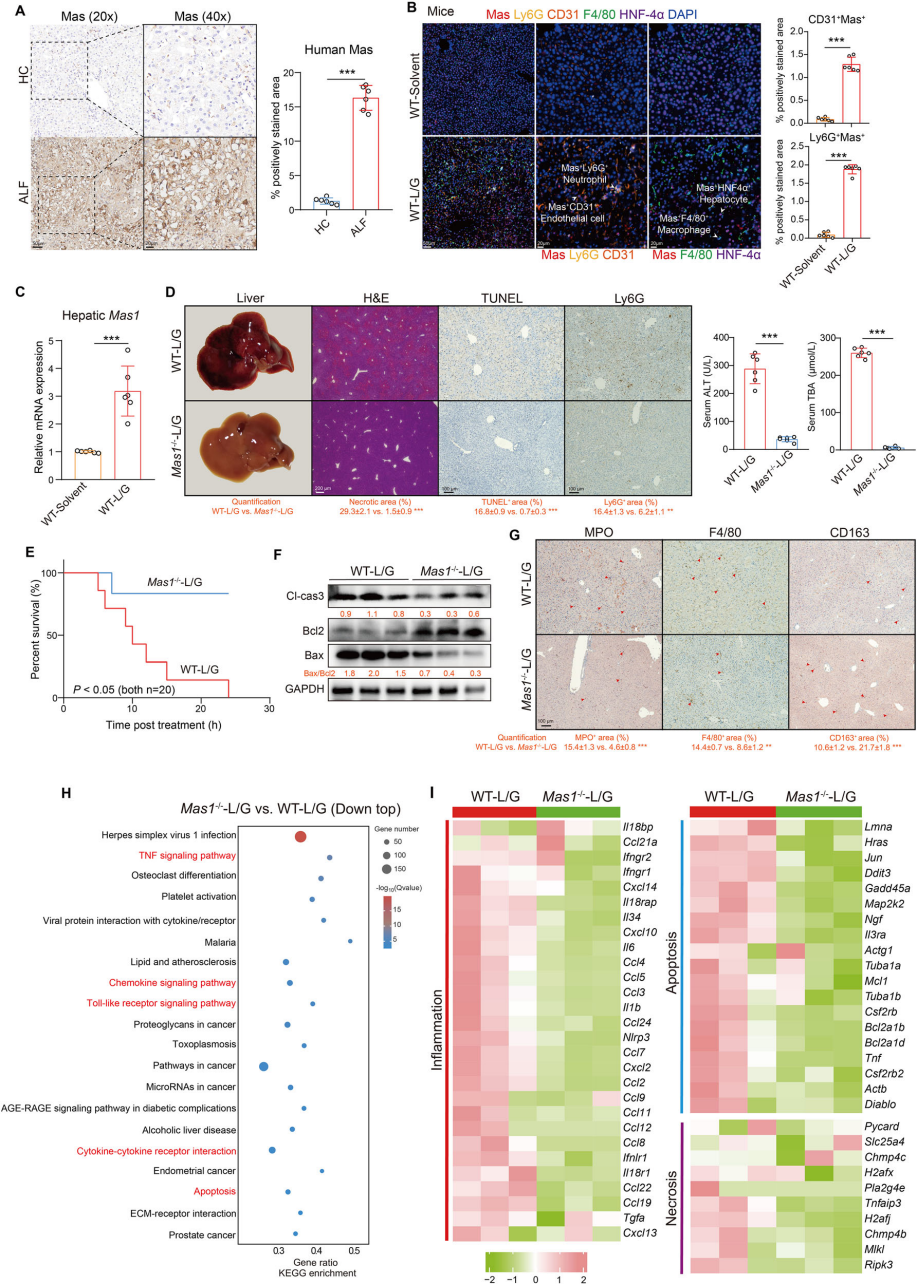
Systemic *Mas1* deficiency protects mice from L/G challenge. A) Representative immunohistochemical staining (left) with the quantification (right) of Mas in HC and ALF patients (*n* = 6 per group, two‐sided Student's *t*‐test, *p* = 3.68 × 10^−4^). Scale bars are shown as indicated. B) Representative mIHC staining of Mas on hepatocytes (HNF4a^+^), endothelial cells (ECs, CD31^+^), macrophages (F4/80^+^) and neutrophils (Ly6G^+^) in liver sections from WT‐solvent and WT‐L/G groups with the quantification (left) of Mas^+^ neutrophils and ECs (two‐sided Student's *t*‐test, *p* = 3.19 × 10^−4^ and *p* = 2.85 × 10^−4^ from top to bottom). Scale bars are shown as indicated. C) Intrahepatic *Mas1* mRNA expression in mice (two‐sided Student's *t*‐test, *p* = 8.12 × 10^−4^). WT and *Mas1* ^−/−^ mice were challenged with a standard dose of L/G or solvent control for 5 h (D&F, G, *n* = 6 per group; H&I, *n* = 3 per group, bulk RNA‐seq). D) Representative liver photographs, and immunohistochemical staining with the quantification (below) of H&E (hemorrhagic necrosis), TUNEL (apoptosis), and Ly6G (neutrophils) in WT‐L/G and *Mas1* ^−/−^‐L/G groups (left, two‐sided Student's *t*‐test, *p* = 2.08 × 10^−4^, *p* = 3.11 × 10^−4^ and *p* = 5.80 × 10^−3^ from left to right). Scale bars are shown as indicated. Serum levels of ALT and TBA (right, two‐sided Student's *t*‐test, *p* = 2.21 × 10^−4^ and *p* = 6.19 × 10^−4^ from left to right). E) Survival curves of WT and *Mas1* ^−/−^ mice treated with a lethal dose of L/G (both *n* = 20, Log‐rank test, *p* = 1.13 × 10^−2^). F) Representative immunoblots of liver tissues with the quantification (below). G) Representative immunohistochemical staining with the quantification (below) of MPO (neutrophils), F4/80 (macrophages) and CD163 (M2 polarization) (two‐sided Student's *t*‐test, *p* = 3.89 × 10^−4^, *p* = 5.59 × 10^−3^ and *p* = 4.64 × 10^−4^ from left to right). H) KEGG pathway enrichment analysis of DEGs in livers from *Mas1* ^−/−^‐L/G and WT‐L/G mice (bulk RNA‐seq). The top 20 down‐regulated pathways are shown. I) Heat maps show the intrahepatic expression of genes enriched in the inflammation, apoptosis and necrosis pathways. Data are presented as mean ± SD (**p < 0.01, ***p < 0.001). See also related Figure S1 (Supporting Information).

Compared to healthy controls (HC), patients with ALF exhibited a significantly higher intrahepatic Mas expression (**Figure** **1**A). The standard dose of L/G challenge induced severe liver damage in mice, characterized by hemorrhagic necrosis, neutrophil infiltration, and elevated serum ALT and TBA levels, resembling human ALF (Figure S1A, B, Supporting Information). In addition, the WT‐L/G group exhibited significantly higher intrahepatic Mas mRNA and protein expression (Figure 1B,C; Figure S1B, Supporting Information). Furthermore, Mas expression was predominantly upregulated in neutrophils and endothelial cells (ECs), showing statistically significant differences (Figure 1B). However, the increase in Mas expression in macrophages and total leukocytes was not statistically significant (Figure S1C, Supporting Information). Therefore, we hypothesized that Mas signaling in ECs and neutrophils contributes significantly to the response during L/G challenge.

### 2.2 Systemic *Mas1* Deficiency Protects Mice from L/G Challenge

To assess the role of Mas, we compared disease phenotypes between WT‐L/G and *Mas1* ^−/−^‐L/G mice. In a notable manner, *Mas1* ^−/−^ mice exhibited markedly reduced liver hemorrhage (H&E), cell death (TUNEL), and neutrophil (Ly6G) infiltration in histology (Figure 1D). They showed significantly lower serum levels of alanine aminotransferase (ALT), indicating reduced liver cell injury, and decreased levels of total bile acid (TBA) and alkaline phosphatase (ALP), suggesting less cholestasis. Additionally, antioxidant enzyme superoxide dismutase (SOD) levels increased (Figure 1D; Figure S1D, Supporting Information). Furthermore, upon exposure to a lethal dose of L/G challenge, *Mas1* ^−/−^ mice demonstrated a significantly higher survival rate compared to WT mice (Figure 1E). Subsequent WB analysis revealed a significant reduction in the intrahepatic protein levels of cell death markers, including cleaved caspase‐3 and the Bax/Bcl‐2 ratio, in the *Mas1* ^−/−^‐L/G group (Figure 1F). The immunohistochemical results indicated that *Mas1* ^−/−^‐L/G mice had reduced intrahepatic neutrophil and macrophage infiltration and increased CD163 expression indicating a shift toward the anti‐inflammatory M2 macrophage phenotype (Figure 1G). Liver transcriptome (bulk RNA‐seq) data revealed that *Mas1* ^−/−^‐L/G mice significantly reduced inflammation and cell death pathways, including apoptosis and necrosis, as indicated by both KEGG pathway (Figure 1H) and gene set (Figure 1I) enrichment analysis. Furthermore, an in vivo assessment was conducted to evaluate the effects of ANG‐(1‐7), an endogenous ligand for the Mas receptor, during L/G challenge. As expected, ANG‐(1‐7) significantly worsened the ALF phenotype induced by a lower dose of L/G exposure, as shown by compromised liver tissue integrity, elevated serum biomarkers, and reduced survival rates (Figure S1E–H, Supporting Information). Conversely, the Mas receptor inhibitor, A779 provided substantial protection against a standard L/G dose in mice (Figure S1I–L, Supporting Information). These findings indicate that *Mas1* ^−/−^ confers a protective effect against L/G‐induced inflammatory responses and cell death.

### 2.3 Systemic *Mas1* Deficiency Reduces NETs Formation in Mice Exposed to L/G

**Figure 2:**
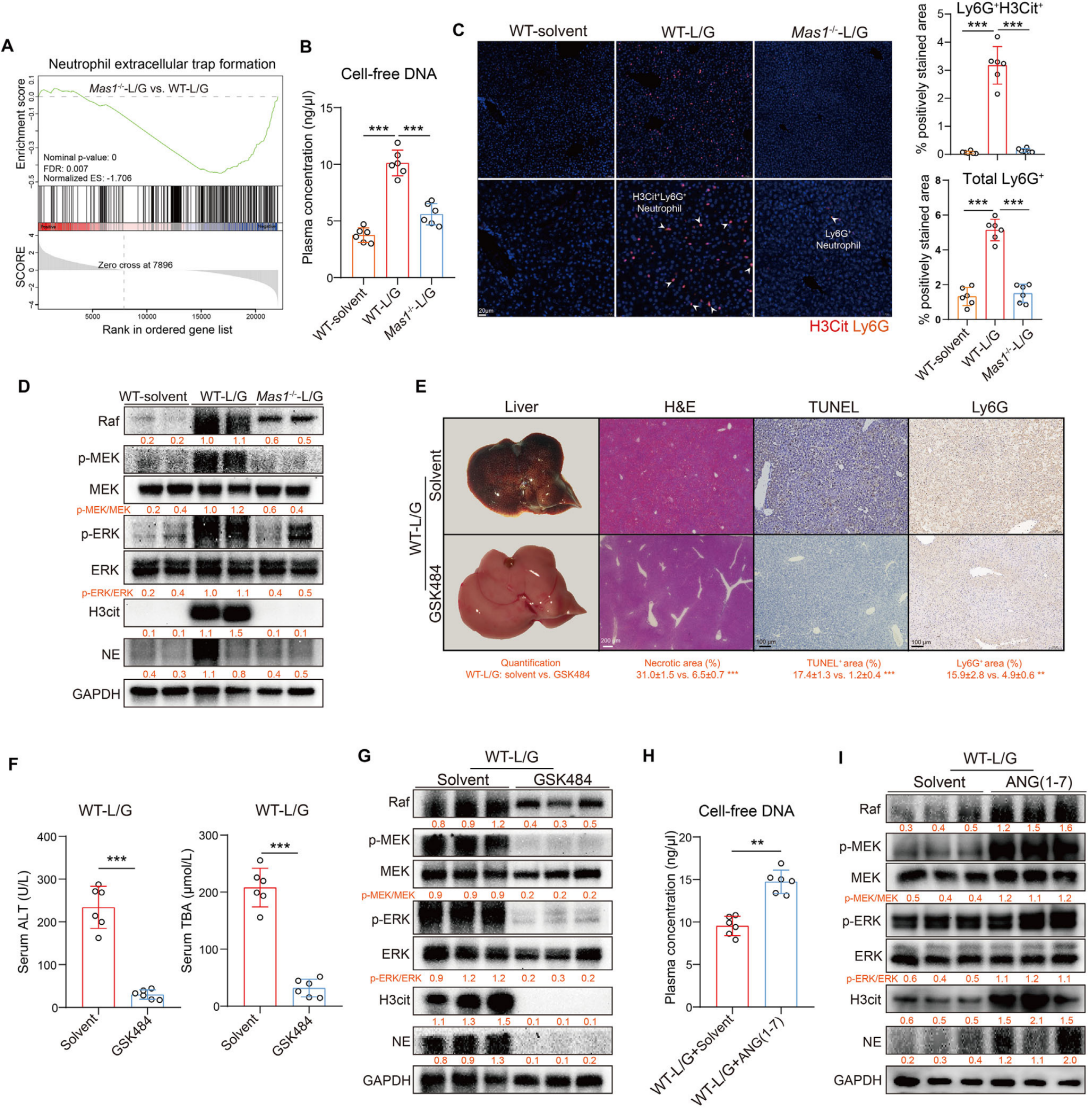
Systemic *Mas1* deficiency reduces NETs formation in mice exposed to L/G. A) GSEA of KEGG enrichment plot in livers from WT‐L/G and *Mas1* ^−/−^‐L/G mice (both *n* = 3, bulk RNA‐seq). B) Plasma levels of cell‐free DNA in WT‐solvent, WT‐L/G, and *Mas1* ^−/−^‐L/G groups (*n* = 6 per group, One‐way ANOVA with Tukey's test, two‐sided Student's *t*‐test, *p* = 5.12 × 10^−4^ and *p* = 2.67 × 10^−4^). C) Representative mIHC staining with the quantification (*n* = 6 per group) of intrahepatic H3Cit^+^ and total neutrophils (One‐way ANOVA with Tukey's test, two‐sided Student's *t*‐test, *p* = 5.69 × 10^−4^, *p* = 3.12 × 10^−4^, *p* = 4.07 × 10^−4^ and *p* = 7.21 × 10^−4^ from left to right, from top to bottom). Scale bar: 20 µm. D) Representative immunoblots (Raf‐MEK‐ERK pathway and markers for NETs formation: H3Cit, NE) of liver tissues with the quantification (below). WT‐L/G mice were prophylactically treated with GSK484 (the NETs inhibitor) or solvent control (*n* = 6 per group, E–G). E) Representative liver photographs and immunohistochemical staining with the quantification (below) of H&E, TUNEL, and Ly6G (two‐sided Student's *t*‐test, *p* = 4.65 × 10^−4^, *p* = 5.30 × 10^−4^ and *p* = 6.02 × 10^−3^ from left to right). Scale bars are shown as indicated. F) Serum levels of ALT and TBA (two‐sided Student's *t*‐test, *p* = 5.30 × 10^−4^ and *p* = 3.27 × 10^−4^ from left to right). G) Representative immunoblots of liver tissues with the quantification (below). WT‐L/G mice were pre‐treated with Ang‐(1‐7) or solvent control (*n* = 6 per group, H,I). H) Plasma levels of cell‐free DNA (two‐sided Student's *t*‐test, *p* = 6.70 × 10^−3^). I) Representative immunoblots of liver tissues with the quantification (below). Data are presented as mean ± SD (**p < 0.01, ***p < 0.001).

The mechanism of hepatic Mas signaling in ALF mice was further explored using bulk RNA‐seq. GSEA revealed that the “NET formation” pathway was significantly downregulated in *Mas1* ^−/−^‐L/G compared to WT‐L/G mice, implying NETs as a potential downstream mechanism of Mas signaling (**Figure** **2**A). *Mas1* ^−/‐^ can attenuate neutrophil infiltration and excessive NETs formation in mice exposed to L/G. This was corroborated by the measurements of serum levels of cell‐free DNA (cfDNA), a critical component of NETs (Figure 2B), and protein levels of Ly6G, citrullinated histone H3 (H3cit), and neutrophil elastase (NE) in the liver (Figure 2C,D). Recent studies show that cfDNA, unlike MPO‐DNA, is closely associated with the prognosis of ALF, ACLF, and sepsis patients, despite both indicating NETs.^[^ ^19^, ^20^, ^21^ ^]^ Therefore, cfDNA was selected instead of MPO‐DNA to evaluate NETs in this study. Previous studies have demonstrated that NETs formation requires activation of the classical mitogen‐activated protein kinase (MAPK) signaling pathway, specifically the Raf/MEK/ERK cascade.^[^ ^22^ ^]^ Hence, WB analysis was employed to investigate the aforementioned pathways, revealing significant down‐regulation of the Raf/MEK/ERK cascade in *Mas1* ^−/−^‐L/G liver samples (Figure 2D). The critical role of NETs in the L/G challenge was substantiated by the in vivo administration of GSK484, a NETs inhibitor, in WT‐L/G mice, which significantly reduced liver inflammation and cell death (Figure 2E–G). Conversely, prophylactic activation of the Mas receptor by ANG‐(1‐7) significantly stimulated the Raf/MEK/ERK pathway in L/G challenged mice, promoting the formation of NETs (Figure 2H,I).

### 2.4 Single‐Cell Annotation of Cldn1^+^CD177^+^ Neutrophils in *Mas1* ^−/−^‐L/G Mice

**Figure 3:**
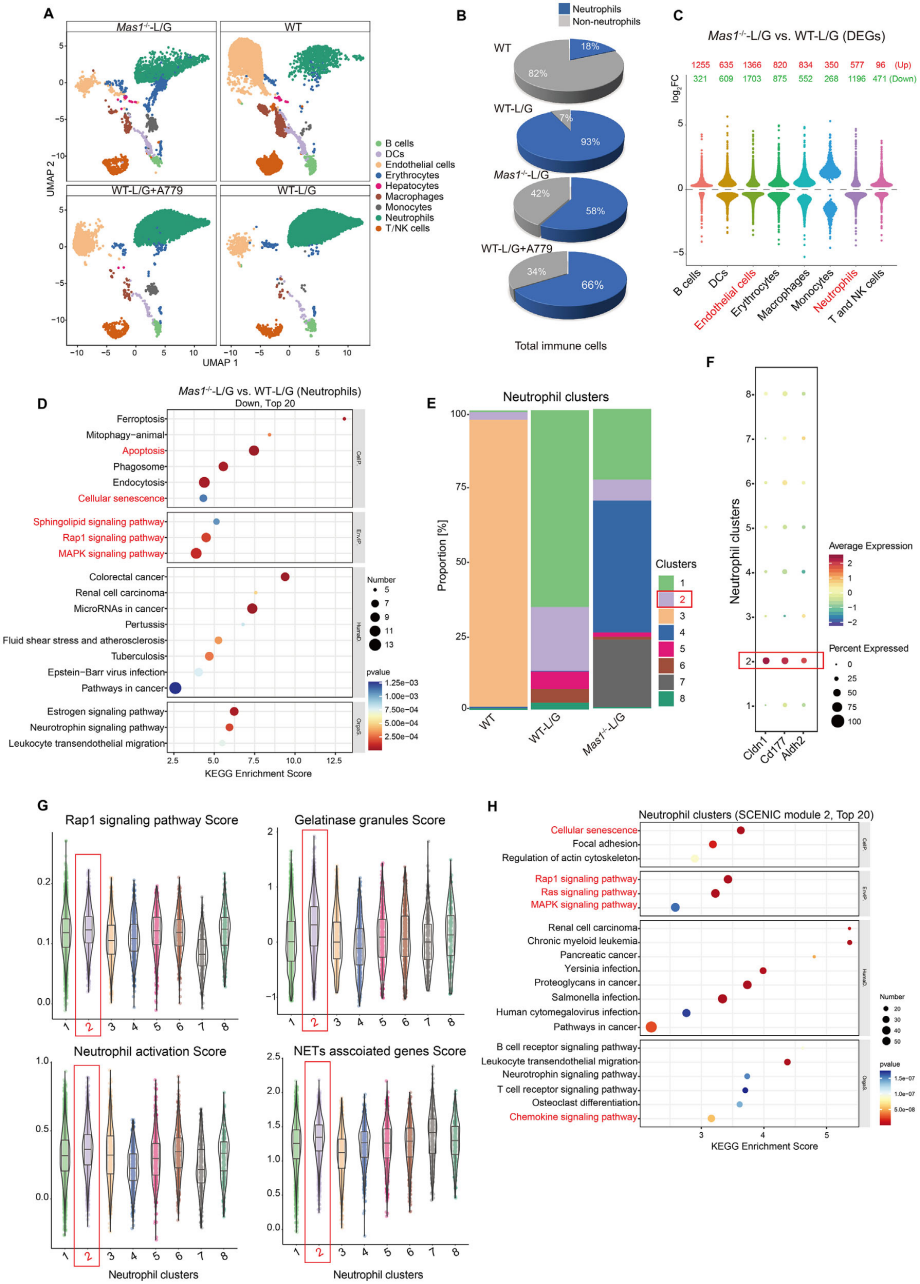
Single‐cell annotation of Cldn1^+^CD177^+^ neutrophils in *Mas1* ^−/−^‐L/G mice. Mouse livers were analyzed by single‐cell RNA‐seq (scRNA‐seq, *n* = 1 per group). A) The uniform manifold approximation and projection (UMAP) plot of main cell types. B) Percentage of neutrophils in total immune cells. C) The DEGs (red, upregulated; green, downregulated) based on cell types in *Mas1* ^−/−^‐L/G versus WT‐L/G group. D) KEGG enrichment of DEGs in neutrophils. Top 20 downregulated are shown in *Mas1* ^−/−^‐L/G versus WT‐L/G. E) Histogram illustrating the distribution of neutrophil cluster proportions across groups. F) Dot plots showing scaled expression of the gene signature for neutrophil cluster 2 (Neu2). G) Violin plots showing the score of indicated pathways for neutrophil clusters. H) Top 20 KEGG enrichment of Module 2 target genes identified by SCENIC analysis of Neu2. See also related Figures S2 and S3 (Supporting Information).

To explore intrahepatic mechanisms related to Mas and NETs formation, scRNA‐seq was used on mouse livers, identifying 16 cell populations across nine major types, resulting in a detailed liver cell atlas (**Figure** **3**A; Figure S2A,B, Supporting Information). Neutrophils, as primary responders and are pivotal immune cells in the context of L/G exposure. The data demonstrated that both the absolute number of neutrophils and their relative proportion among the total immune cells were elevated after L/G challenge, a phenomenon that was attenuated by systemic deficiency of Mas receptor (Figure 3B; Figure S2C, Supporting Information). The differentially expressed genes (DEGs) with significant changes were mainly found in neutrophils and ECs (Figure 3C). Neutrophils in *Mas1* ^−/−^‐L/G versus WT‐L/G group exhibited a remarkable downregulation of signaling pathways implicated in apoptosis, cellular senescence, sphingolipid metabolism, Rap1 and MAPK signaling (Figure 3D), as well as the formation of NETs (Figure S2D, Supporting Information). Further analysis of neutrophil clusters indicated a significant increase in neutrophil cluster 2 (Neu2) after L/G challenge, but a notable decrease was observed in the context of systemic *Mas1* deficiency (Figure 3E; Figure S2E, Supporting Information). Although Neu1 had a higher absolute count than Neu2, the decrease in Neu2 percentage in the *Mas1* ^−/−^‐L/G compared to WT‐L/G group was relatively larger than that of Neu1. Further, scRNA‐seq data revealed that Neu2's Top10 markers were more specific, and functional analysis indicated a significant enrichment of the neutrophil degranulation pathway in Neu2, linking it more closely to NETs formation (data not shown). Thus, we selected Neu2 over Neu1 for further study. The top 3 marker genes of Neu2 are *Cldn1*, *Cd177*, and *Aldh2* (Figure 3F). Consistent with previous studies, Neu2 showed a relatively higher score for “Rap1 signaling, gelatinase granules, neutrophil activation, and NET‐related genes” compared to other neutrophil clusters (Figure 3G), suggesting its crucial role in NETs formation. Understanding transcription networks in ALF is crucial,^[^ ^23^ ^]^ so SCENIC analysis was performed to investigate transcription factors in neutrophil clusters. The highly active regulons in Neu2 consist of *Fosb*, *Nfe2*, *Jund*, and *Hif1a* (Figure S2F, Supporting Information). Further analysis of these regulons using the connection specificity index (CSI) revealed that Neu2 was mainly enriched in Module 2 (Figure S2G, Supporting Information). KEGG enrichment analysis of the target genes within Module 2 revealed that cellular senescence along with Rap1‐, Ras‐, MAPK‐, and chemokine signaling pathways, ranked among the top 20 pathways (Figure 3H). These findings suggest that Neu2 predisposes NETs formation at the transcription factor level. In the pseudotime analysis, Neu2 was positioned at the starting point and exhibited an abundance of genes associated with the Sphingolipid signaling pathway, Sphingolipid metabolism, Cellular senescence, Bile secretion, and Rap1 signaling pathway (Figure S3A,B, Supporting Information). This indicates the roles of sphingolipids and bile components in NETs formation. Additionally, Neu2 significantly upregulated signaling pathways linked to cellular senescence, apoptosis, MAPK, and TNF throughout its developmental trajectory (Figure S3C, Supporting Information).

### 2.5 Col4a1^+^ ECs Trigger NETs Formation in Cldn1^+^CD177^+^ Neutrophils via Col4a1‐Cd44 Interaction

**Figure 4:**
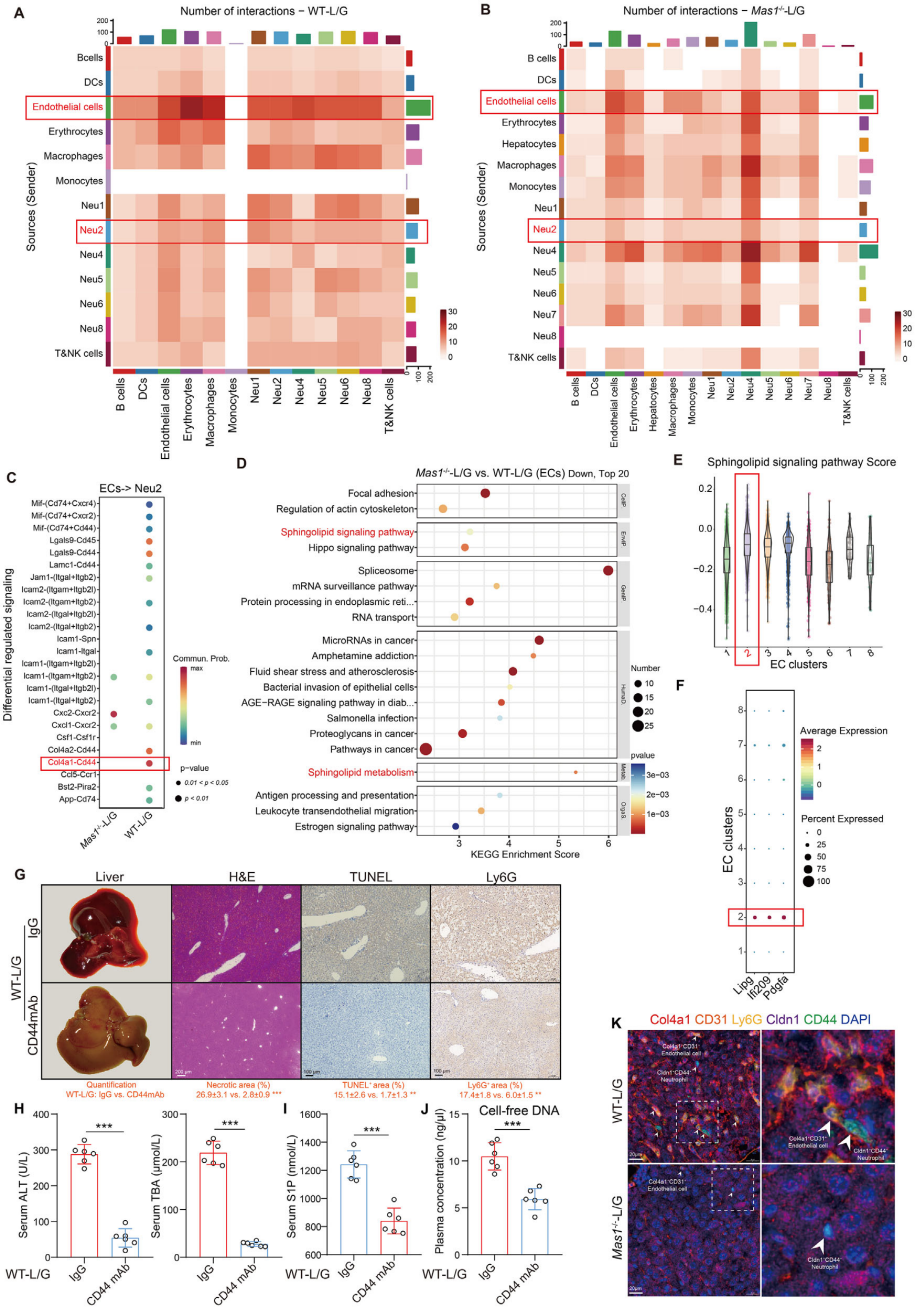
Col4a1^+^ ECs trigger NETs formation in Cldn1^+^CD177^+^ neutrophils via Col4a1‐CD44 interaction. A,B) Heatmap shows the number of interactions between neutrophil clusters and other cell types in the WT‐LG (A) and *Mas1* ^−/−^‐L/G (B) groups. C) Bubble plots display the likelihood of ligand‐receptor pairs for ECs signaling to Neu2 in both WT‐LG and *Mas1* ^−/−^‐L/G mice. D) KEGG enrichment of DEGs in ECs. Top 20 downregulated are shown in *Mas1* ^−/−^‐L/G versus WT‐L/G. E) Violin plots illustrate the score of sphingolipid signaling pathway for EC clusters. F) Dot plots show scaled expression of the gene signature for EC cluster 2 (EC2). WT‐L/G mice were pre‐treated with CD44 mAb or IgG control (*n* = 6 per group, G–J). G) Representative liver photographs and immunohistochemical staining with the quantification (below) of H&E, TUNEL, and Ly6G (two‐sided Student's *t*‐test, *p* = 7.82 × 10^−4^, *p* = 4.51 × 10^−3^ and *p* = 6.06 × 10^−3^ from left to right). Scale bars are shown as indicated. H) Serum levels of ALT and TBA (two‐sided Student's *t*‐test, *p* = 3.98 × 10^−4^ and *p* = 6.15 × 10^−4^ from left to right). I) Serum concentrations of S1P (two‐sided Mann–Whitney U test, *p* = 8.21 × 10^−4^). J) Plasma levels of cell‐free DNA (two‐sided Student's *t*‐test, *p* = 4.31 × 10^−4^). K) Representative mIHC staining of intrahepatic Col4a1^+^ ECs and Cldn1^+^CD44^+^ neutrophils in WT‐LG and *Mas1* ^−/−^‐L/G mice. Scale bar: 20 µm. Data are presented as mean ± SD or median ± IQR (***p < 0.001, **p < 0.01). See also related Figures S4–S6 (Supporting Information).

Cell Chat analysis of scRNA‐seq data was used to investigate cell communication in the intrahepatic inflammatory microenvironment exposed to L/G.The analysis revealed that *Mas1* ^−/−^ substantially reduced the number and strength of interactions between ECs and Neu2 within the context of L/G challenge (**Figure** **4**A,B; Figure S4A, B, Supporting Information), indicating a potential role of ECs in the NETs formation. Visualization of communication dynamics across various signaling pathways, the collagen signaling pathway involving the ligand‐receptor pair *Col4a1*‐*Cd44* was found to mediate interactions between ECs and Neu2, which is highly active in WT‐L/G mice and its activity was significantly reduced in *Mas1* ^−/−^‐L/G mice (Figure 4C; Figure S4C,D, Supporting Information). As the second‐largest cell population within hepatic tissue, ECs play a critical role in recruiting inflammatory cells because of their unique position at the interface between the liver and bloodstream. Subsequently, scRNA‐seq data was used to perform a comprehensive analysis of ECs and found that sphingolipid metabolism and signaling pathways were significantly downregulated in *Mas1* ^−/−^‐L/G compared to those in WT‐L/G mice (Figure 4D), whereas they were significantly upregulated in WT‐L/G compared to those in WT mice (Figure S4E, Supporting Information). These findings suggest a link between sphingolipid metabolism in ECs and the L/G challenge, with Mas playing a regulatory role in this process. Furthermore, we analyzed ECs by clusters and found that EC2 was the most prevalent cluster in mice after L/G challenge, and its numbers decreased in the *Mas1* ^−/−^‐L/G group (Figure S4F,G, Supporting Information). Notably, only EC2 and EC7 appeared in the *Mas1* ^−/−^‐L/G and WT‐L/G groups, but EC7's count was too low for detailed analysis. EC2 showed a high score for sphingolipid metabolism, with Lipg, Ifi209, and Pdgfa as its top marker genes (Figure 4E,F). SCENIC analysis further identified *Cebpb*, *Myc*, *Irf7*, and *Stat1* as highly active regulons in EC2 primarily linked to inflammation and enriched in Module 2 (Figure S4H,I, Supporting Information). Reactome enrichment analysis of the target genes highlighted their key roles in “neutrophil degranulation” and “cytokine signaling within the immune system” (Figure S5A, Supporting Information). Analysis with scMetabolism_KEGG revealed that EC2 and non‐EC2 have opposing metabolic traits, indicating differing roles of L/G exposure (Figure S5B, Supporting Information). Notably, the “primary bile acid synthesis” pathway is enriched in EC2, and the “histone H3‐R26 citrullination pathway” was significantly upregulated, linking it to bile acid and NETs formation (Figure S5B,C, Supporting Information). In the pseudotime analysis, EC2 was positioned at the beginning, showing marked upregulation of genes involved in “Rap1 signaling,” “Sphingolipid signaling,” “Primary bile acid biosynthesis,” and “Apoptosis” pathways, indicating their potential roles in NETs formation (Figure S6A–D, Supporting Information). The Col4a1‐CD44 interaction is essential for EC2 and Neu2 cell interactions, enhancing Raf/MEK/ERK‐dependent NETs formation in vivo, as shown by administering CD44mAb to WT‐L/G mice (Figure 4G–J, Figure S6E, Supporting Information). Finally, mIHC analysis showed that in WT‐L/G mice, Col4a1^+^ ECs were more frequently found with Cldn1^+^CD44^+^ neutrophils, but this colocalization was notably decreased in *Mas1* ^−/−^‐L/G mice (Figure 4K; Figure S6F, Supporting Information).

### 2.6 Systemic *Mas1* Deficiency Inhibits S1P‐Induced NETs Formation

**Figure 5:**
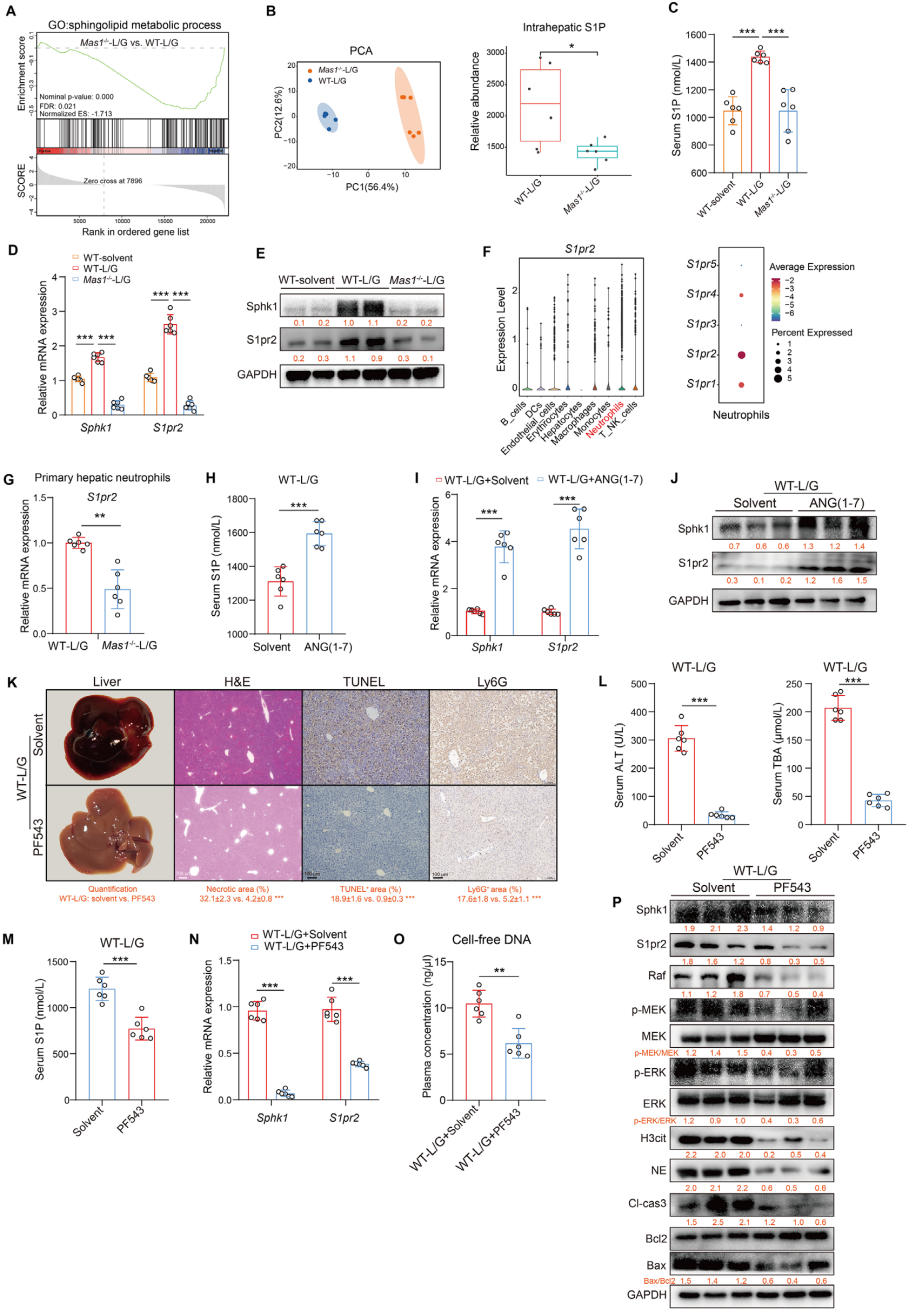
Systemic *Mas1* deficiency inhibits S1P‐induced NETs formation. A) GSEA of GO enrichment plot in livers from WT‐L/G and *Mas1* ^−/−^‐L/G mice (both *n* = 3, bulk RNA‐seq). B) PCA of liver metabolomics (*n* = 6 per group, left) and the relative abundance of intrahepatic S1P (right) (two‐sided Mann–Whitney U test, *p* = 2.58 × 10^−2^). C) Serum S1P levels (all, *n* = 6, One‐way ANOVA with Tukey's test, two‐sided Student's *t*‐test, *p* = 4.28 × 10^−4^ and *p* = 3.27 × 10^−4^ from left to right). D) Intrahepatic mRNA expression of *Sphk1* and *S1pr2* (all, *n* = 6, One‐way ANOVA with Tukey's test, two‐sided Student's *t*‐test, *p* = 3.12 × 10^−4^, *p* = 5.06 × 10^−4^, *p* = 4.18 × 10^−4^ and *p* = 3.91 × 10^−4^ from left to right). E) Representative immunoblots of liver tissues with the quantification (below). F) The violin boxplot (left) illustrates *S1pr2* expression across cell types, and the dot plot (right) presents *S1pr1* through *S1pr5* expression in neutrophils (scRNA‐seq). G) The mRNA expression of *S1pr2* in hepatic neutrophils (both *n* = 6, two‐sided Student's *t*‐test, *p* = 8.12 × 10^−3^). WT‐L/G mice were pre‐treated with Ang‐(1‐7) or solvent control (*n* = 6 per group, H–J). H) Serum S1P levels (two‐sided Mann–Whitney U test, *p* = 4.08 × 10^−4^). I) Intrahepatic mRNA expression of *Sphk1* and *S1pr2* (two‐sided Student's *t*‐test, *p* = 8.17 × 10^−4^ and *p* = 6.35 × 10^−4^ from left to right). J) Representative immunoblots of liver tissues with the quantification (below). WT‐L/G mice were pre‐treated with PF543 (SphK1 inhibitor) or solvent control (*n* = 6 per group, K–P). K) Representative liver photographs and immunohistochemical staining with the quantification (below) of H&E, TUNEL, and Ly6G (two‐sided Student's *t*‐test, *p* = 5.60 × 10^−4^, *p* = 3.19 × 10^−4^ and *p* = 8.72 × 10^−4^ from left to right). Scale bars are shown as indicated. L) Serum levels of ALT and TBA (two‐sided Student's *t*‐test, *p* = 3.96 × 10^−4^ and *p* = 4.18 × 10^−4^ from left to right). M) Serum levels of S1P (two‐sided Student's *t*‐test, *p* = 2.98 × 10^−4^). N) Intrahepatic mRNA expression of *Sphk1* and *S1pr2* (two‐sided Student's *t*‐test, *p* = 7.61 × 10^−4^ and *p* = 3.59 × 10^−4^ from left to right). O) Plasma levels of cell‐free DNA (two‐sided Mann–Whitney U test, *p* = 3.18 × 10^−3^). P) Representative immunoblots of liver tissues with the quantification (below). Data are presented as mean ± SD or median ± IQR (*p < 0.05, **p < 0.01, ***p < 0.001). See also related Figure S7 (Supporting Information).

S1P, generated by sphingosine kinases during membrane sphingolipid metabolism, acts as a crucial signaling molecule that can exit the cell and activate S1P receptors (S1PRs), thereby triggering pathways such as Raf/MEK/ERK and PI3K/Akt. S1PR2 is crucial for neutrophil migration, activation, and inflammation, with studies showing it is more highly expressed in neutrophils than other S1PR subtypes. Its expression is regulated by inflammatory signals and chemokines, highlighting its importance in neutrophil recruitment.^[^ ^24^ ^]^ First, GSEA of liver RNA‐seq data revealed a significant reduction in the sphingolipid metabolic process in *Mas1* ^−/−^‐L/G mice (**Figure** **5**A), which aligned with lower levels of S1P detected in both liver and serum using non‐targeted metabolomics (Figure 5B) and ELISA (Figure 5C) methods. Besides, the mRNA and protein levels of SphK1 and S1PR2 were significantly downregulated in the *Mas1* ^−/−^‐L/G group (Figure 5D,E). Using scRNA‐seq, we conducted a systematic evaluation of *S1pr2* expression across a variety of cell types, as well as the expression of *S1pr1* through *S1pr5* specifically in neutrophils, thereby confirming that *S1pr2* is predominantly expressed in neutrophils (Figure 5F; Figure S7, Supporting Information). Additionally, *S1pr2* expression in neutrophils isolated from the livers of *Mas1* ^−/−^‐L/G mice was significantly lower than that in the control group (Figure 5G). Furthermore, compared to *Mas1* ^−/−^, ANG‐(1‐7) had an opposite effect serum S1P levels, as well as hepatic *Sphk1* and *S1pr2* mRNA and protein (Figure 5H–J). The SphK1 inhibitor PF543 reduced S1P production and S1PR2 levels, significantly inhibiting Raf/MEK/ERK‐dependent NETs formation and improving ALF (Figure 5K–P). Further exploration was conducted to uncover how the Mas receptor influences sphingolipid signaling.

### 2.7 Systemic Mas Receptor Modulates Gut Microbiota and Deoxycholic Acid (DCA) Production in Mice After L/G Challenge

**Figure 6:**
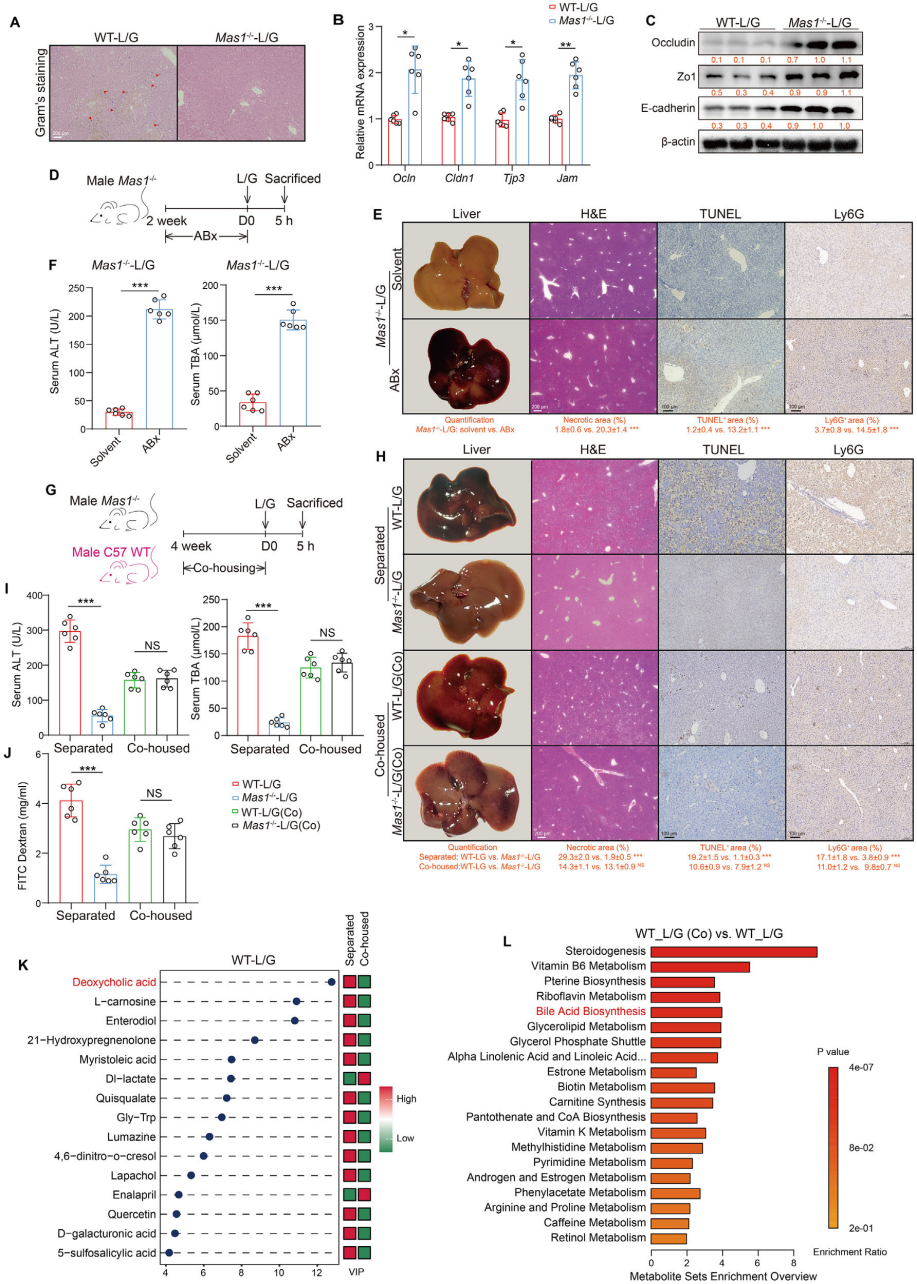
Systemic Mas modulates gut microbiota and DCA production in mice after L/G challenge. A) Gram's staining of liver sections (red arrows indicate positive staining). Scale bar: 200 µm. B,C) The mRNA expression (B) (both *n* = 6, two‐sided Student's *t*‐test, *p* = 2.12 × 10^−2^, *p* = 3.10 × 10^−2^, *p* = 2.68 × 10^−2^ and *p* = 6.52 × 10^−3^ from left to right) and representative immunoblots (C, the quantification, below) of markers for mucosal barrier integrity in the ileum. *Mas1* ^−/−^‐L/G mice were pre‐treated with oral antibiotics (ABx) or solvent control (*n* = 6 per group, D–F). D) The timeline for ABx treatment. E) Representative liver photographs and immunohistochemical staining with the quantification of H&E, TUNEL and Ly6G (two‐sided Student's *t*‐test, *p* = 6.50 × 10^−4^, *p* = 7.92 × 10^−4^ and *p* = 3.41 × 10^−4^ from left to right). Scale bars are shown as indicated. F) Serum levels of ALT and TBA (two‐sided Student's *t*‐test, *p* = 6.93 × 10^−4^ and *p* = 4.85 × 10^−4^ from left to right). *Mas1* ^−/−^ and WT mice were co‐housed or separated for 4 weeks before L/G challenge. (*n* = 6 per group, G–L). G) The timeline for co‐housing experiments. H) Representative liver photographs and immunohistochemical staining with quantification of H&E, TUNEL and Ly6G (two‐sided Student's *t*‐test, Separated: *p* = 8.71 × 10^−4^, *p* = 5.93 × 10^−4^ and *p* = 6.38 × 10^−4^ from left to right; Co‐housed: *p* = 0.99, *p* = 0.68 and *p* = 0.81 from left to right). Scale bars are shown as indicated. I) Serum levels of ALT, TBA (One‐way ANOVA with Tukey's test, two‐sided Student's *t*‐test, *p* = 3.29 × 10^−4^, *p* = 0.76, *p* = 7.11 × 10^−4^ and *p* = 0.39 from left to right). J) In vivo assay of intestinal permeability (lower panel, One‐way ANOVA with Tukey's test, two‐sided Student's *t*‐test, *p* = 8.02 × 10^−4^ and *p* = 0.68 from left to right). K) VIP plot of top 15 differential intestinal metabolites (MS2 level) in the co‐housed and separated WT‐L/G mice. L) MSEA plot of top 20 downregulated pathways in co‐housed versus separated WT‐L/G mice. Data are presented as mean ± SD or median ± IQR (*p < 0.05, **p < 0.01, ***p < 0.001). See also related Figure S8 (Supporting Information).

ALF is characterized by severe liver damage caused by endotoxemia originating from the intestines, highlighting the critical role of the gut microbiota in its pathogenesis. First, Gram‐positive bacteria were detected in the livers of WT‐L/G mice concurrent with intestinal mucosal barrier damage; this damage was mitigated in the *Mas1* ^−/−^‐L/G group (**Figure** **6**A). Second, the enhancement of intestinal mucosal barrier function in *Mas1* ^−/−^ mice was evidenced by the upregulation of mRNA and protein expression of key intestinal barrier markers (Figure 6B,C). In addition, a 2‐week antibiotic (ABx) gavage experiment showed that ABx treatment eliminated the gut bacteria and worsened the disease phenotype in *Mas1* ^−/−^‐L/G mice (Figure 6D–F). In contrast, ABx had no significant effect on liver inflammation, cell death, or NETs formation in WT‐L/G mice (Figure S8A–C, Supporting Information). These results underscore the crucial role of the gut microbiota in the protective effects of Mas signaling. Moreover, co‐housing WT with *Mas1* ^−/−^ mice for 4 weeks before the L/G challenge resulted in a greater similarity in their phenotypic characteristics (Figure 6G–I). Specifically, liver injury improved in the WT‐L/G group after co‐housing, whereas *Mas1* ^−/−^‐L/G mice showed a slight worsening. Following the co‐housing intervention, the cfDNA levels in both groups became comparable, showing no statistically significant differences. Notably, plasma cfDNA levels in WT‐L/G mice decreased post co‐housing compared to pre‐co‐housing levels (Figure S8D, Supporting Information). Additionally, the intestinal permeability assay revealed that *Mas1* ^−/−^ mice maintained the integrity of the intestinal barrier; however, this protection decreased after co‐housing (Figure 6J). Further fecal metabolomics analysis identified DCA as the Top 1 downregulated metabolite in co‐housed WT‐L/G mice, which is a well‐established inhibitor of farnesoid X receptor (FXR) (Figure 6K). This finding may be related to improved liver injury, as the bile acid synthesis pathway was significantly downregulated in WT mice after co‐housing (Figure 6L).

### 2.8 FXR Serves as the Pivotal Upstream Regulator of S1P in L/G Challenge

**Figure 7:**
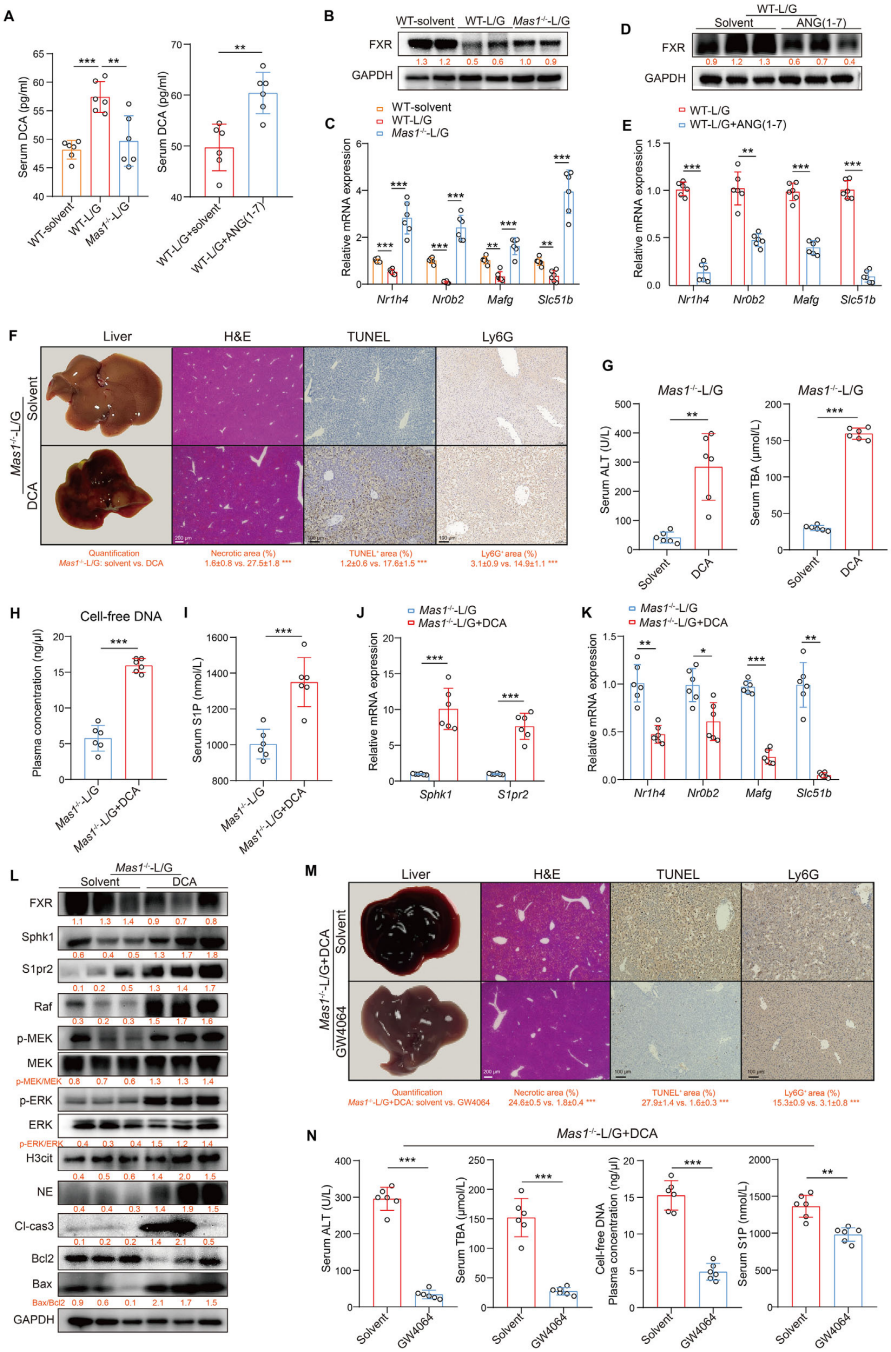
FXR serves as the pivotal upstream regulator of S1P in L/G challenge. A) Serum levels of DCA (all, *n* = 6, One‐way ANOVA with Tukey's test, two‐sided Student's *t*‐test, *p* = 7.29 × 10^−4^, *p* = 4.74 × 10^−3^ and *p* = 5.69 × 10^−3^ from left to right). B) Representative immunoblots of liver tissues with the quantification (below). C) The intrahepatic mRNA expression of FXR and its target genes in WT, WT‐L/G and *Mas1* ^−/−^‐L/G mice (all, *n* = 6, One‐way ANOVA with Tukey's test, two‐sided Student's *t*‐test, *p* = 6.58 × 10^−4^, *p* = 3.81 × 10^−4^, *p* = 2.39 × 10^−4^, *p* = 4.01 × 10^−4^, *p* = 4.96 × 10^−3^, *p* = 8.23 × 10^−4^, *p* = 5.61 × 10^−3^ and *p* = 3.49 × 10^−4^ from left to right). D) Representative immunoblots of liver tissues with the quantification (below). E) The intrahepatic mRNA expression of FXR and its target genes (both, *n* = 6, two‐sided Student's *t*‐test, *p* = 8.04 × 10^−4^, *p* = 3.71 × 10^−3^, *p* = 4.18 × 10^−4^ and *p* = 5.69 × 10^−4^ from left to right). *Mas1* ^−/−^‐L/G mice were pre‐treated with oral DCA or solvent control (*n* = 6 per group, F–L). F) Representative liver photographs and immunohistochemical staining with quantification of H&E, TUNEL and Ly6G (two‐sided Student's *t*‐test, *p* = 5.39 × 10^−4^, *p* = 3.86 × 10^−4^ and *p* = 4.92 × 10^−4^ from left to right). Scale bars are shown as indicated. G) Serum levels of ALT and TBA (two‐sided Student's *t*‐test, *p* = 3.47 × 10^−3^ and *p* = 5.61 × 10^−4^ from left to right). H) Plasma levels of cell‐free DNA (two‐sided Mann–Whitney U test, *p* = 3.91 × 10^−4^). I) Serum levels of S1P (two‐sided Student's *t*‐test, *p* = 4.65 × 10^−4^). J) Intrahepatic mRNA expression of *Sphk1* and *S1pr2* (two‐sided Student's *t*‐test, *p* = 4.37 × 10^−4^ and *p* = 3.42 × 10^−4^ from left to right). K) The intrahepatic mRNA expression of FXR and its target genes (two‐sided Student's *t*‐test, *p* = 7.92 × 10^−3^, *p* = 4.90 × 10^−2^, *p* = 6.03 × 10^−4^ and *p* = 3.95 × 10^−3^ from left to right). L) Representative immunoblots of liver tissues with the quantification (below). *Mas1* ^−/−^‐DCA‐L/G mice were pre‐treated with oral FXR agonist GW4064 or solvent control (*n* = 6 per group, M,N). M) Representative liver photographs and immunohistochemical staining with quantification of H&E, TUNEL and Ly6G (two‐sided Student's *t*‐test, *p* = 3.25 × 10^−4^, *p* = 4.97 × 10^−4^ and *p* = 8.21 × 10^−4^ from left to right). Scale bars are shown as indicated. N) Serum levels of ALT and TBA (two‐sided Student's *t*‐test, *p* = 3.12 × 10^−4^ and *p* = 5.98 × 10^−4^ from left to right). Plasma levels of cell‐free DNA (two‐sided Student's *t*‐test, *p* = 4.65 × 10^−4^). Serum levels of S1P (two‐sided Student's *t*‐test, *p* = 8.71 × 10^−3^). Data are presented as mean ± SD or median ± IQR (*p < 0.05, **p < 0.01, ***p < 0.001). See also related Figure S9 (Supporting Information).

Given the established link between DCA and FXR signaling, we then examined FXR and its target genes in *Mas1* ^−/−^‐L/G mice. The results showed a significant decrease in serum DCA concentration in *Mas1* ^−/−^‐L/G mice compared to WT‐L/G mice (**Figure** **7**A) and confirmed substantial activation of the liver FXR signaling pathway, as evidenced by the higher mRNA and protein expression levels of FXR (*Nr1h4*) and its target genes (*Mafg*, *Nr0b2*, and *SIc51b*) (Figure 7B,C). Conversely, in vivo pretreatment with ANG‐(1‐7) significantly inhibited FXR signaling in the liver of WT‐L/G mice (Figure 7D,E). These findings suggest that Mas serves as a crucial upstream regulator of FXR signaling during the L/G challenge. Previous study has indicated that FXR is a key regulator of SphK1, and its activation inhibits SphK1 activity.^[^ ^25^ ^]^ Hence, we orally administered DCA to *Mas1* ^−/−^ mice for 2 weeks before the L/G challenge and observed that complete blockade of FXR signaling significantly abolished the protective effect in *Mas1* ^−/‐^ (Figure 7F,G). Furthermore, DCA significantly activated SphK1, leading to an increase in the serum S1P level, which acts through S1PR2 and triggers the formation of Raf/MEK/ERK‐dependent NETs, resulting in increased liver inflammation and cellular damage (Figure 7H–L). In addition, using *Mas1* ^−/−^‐L/G mice treated with DCA, we administered the FXR agonist GW4064 and observed that it significantly alleviated DCA‐induced liver inflammation and cellular damage in *Mas1* ^−/−^‐L/G mice. This effect was associated with reduced S1P production and subsequent NETs formation (Figure 7M,N; Figure S9A, Supporting Information). In *Mas1* ^−/−^‐L/G mice, we also showed that the oral FXR antagonist ZGG worsened ALF and boosted S1P‐dependent NETs formation (Figure S9B,C, Supporting Information). These findings supported the hypothesis that DCA‐induced S1P production depends on FXR inhibition.

### 2.9 Intrahepatic SHP2 Functions Downstream of Mas Receptor to Regulate the FXR‐S1P‐NETs Axis During L/G Challenge

**Figure 8:**
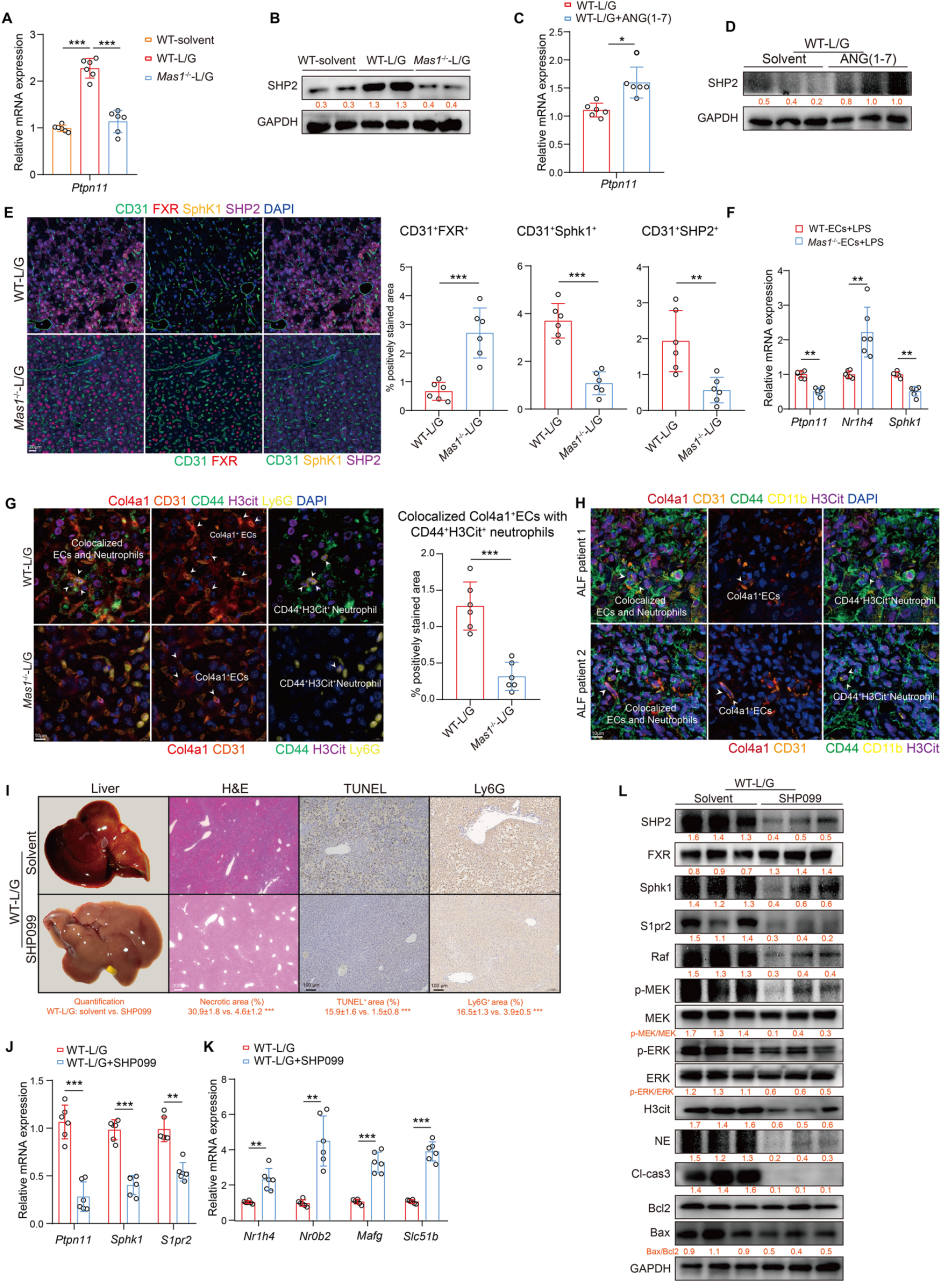
Intrahepatic SHP2 functions downstream of Mas to regulate the FXR‐S1P‐NETs axis during L/G challenge. A) Intrahepatic mRNA expression of *Ptpn11* in WT‐Solvent, WT‐L/G and *Mas1* ^−/−^‐L/G groups (all, *n* = 6; One‐way ANOVA with Tukey's test, two‐sided Student's *t*‐test, *p* = 6.01 × 10^−4^ and *p* = 3.94 × 10^−4^ from left to right). B) Representative liver immunoblots of SHP2 with the quantification (below). C) Intrahepatic mRNA expression of *Ptpn11* (both, *n* = 6, two‐sided Student's *t*‐test, *p* = 3.12 × 10^−2^). D) Representative liver immunoblots of SHP2 with the quantification (below). E) Representative mIHC staining of FXR, SphK1, and SHP2 on CD31^+^ ECs in liver sections with the quantification (two‐sided Student's *t*‐test, *p* = 3.19 × 10^−4^, *p* = 2.37 × 10^−4^ and *p* = 4.73 × 10^−3^ from left to right). Scale bars are shown as indicated. F) The mRNA expression of *Ptpn11*, *Nr1h4*, and *Sphk1* (two‐sided Student's *t*‐test, *p* = 7.62 × 10^−3^, *p* = 5.08 × 10^−3^ and *p* = 7.69 × 10^−3^ from left to right). G‐H) Representative mIHC staining of co‐localization between intrahepatic Col4a1^+^ ECs and H3Cit^+^CD44^+^ neutrophils in ALF mice (G) and patients (H); and the quantification (right in G; two‐sided Student's *t*‐test, *p* = 5.78 × 10^−4^). Scale bars are shown as indicated. WT mice were treated with SHP099 (SHP2 inhibitor) or solvent control before L/G challenge (*n* = 6 per group, I‐L). I) Representative liver photographs and immunohistochemical staining with the quantification (below) of H&E, TUNEL, and Ly6G (two‐sided Student's *t*‐test, *p* = 2.94 × 10^−4^, *p* = 3.47 × 10^−4^ and *p* = 5.49 × 10^−4^ from left to right). Scale bars are shown as indicated. J) Intrahepatic mRNA expression of *Ptpn11*, *Sphk1* and *S1pr2* (two‐sided Student's *t*‐test, *p* = 5.46 × 10^−4^, *p* = 3.94 × 10^−4^ and *p* = 3.68 × 10^−3^ from left to right). K) Intrahepatic mRNA expression of FXR and its target genes (two‐sided Student's *t*‐test, *p* = 7.61 × 10^−3^, *p* = 3.86 × 10^−3^, *p* = 6.72 × 10^−4^ and *p* = 4.90 × 10^−4^ from left to right). L) Representative immunoblots of liver tissues with the quantification (below). Data are presented as mean ± SD (*p < 0.05, **p < 0.01, ***p < 0.001). See also related Figure S10 (Supporting Information).

SHP2, encoded by the *Ptpn11* gene, is a protein tyrosine phosphatase containing the Src homology 2 domain and is known as a downstream molecule of the Mas receptor. Previous studies have indicated that SHP2 is a key signaling regulator that directly inhibits FXR and its target genes, thereby affecting bile acid synthesis in the liver.^[^ ^26^ ^]^ Therefore, we propose that Mas influences the FXR‐S1P‐NETs axis via SHP2 during the L/G challenge. Our investigations initially revealed a significant increase in SHP2 mRNA and protein levels in WT mice after L/G exposure, which can be attenuated by *Mas1* ^−/‐^ (**Figure** **8**A,B). Additionally, ANG‐(1‐7) increased the expression of SHP2 in WT‐L/G mice (Figure 8C,D). To investigate the regulatory role of Mas signaling in the SHP2‐FXR‐S1P axis in ECs following L/G challenge, we initially evaluated the expression levels of *Ptpn11*, *Nr1h4*, and *Sphk1* in various cell types using scRNA‐seq. Our analysis revealed that these genes were predominantly expressed in ECs (Figure S10A, Supporting Information). Next, we evaluated the protein expression levels of FXR, SHP2, and SphK1 in ECs using mIHC. The results demonstrated that, compared with the WT‐L/G group, FXR protein level in ECs of the *Mas1* ^−/−^‐L/G group was significantly elevated, whereas the expression levels of SHP2 and SphK1 were markedly reduced (Figure 8E). Furthermore, primary hepatic ECs were isolated from WT and *Mas1* ^−/‐^ mice and subjected to in vitro LPS stimulation. *Mas1* ^−/−^ had significantly reduced *Ptpn11* and *Sphk1* mRNA levels and increased *Nr1h4* mRNA levels in ECs (Figure S10B, Supporting Information; Figure 8F). We used mIHC to demonstrate that the interaction between Col4a1^+^ ECs and CD44^+^H3Cit^+^ neutrophils was significantly decreased in the livers of *Mas1* ^−/−^‐L/G mice compared to WT‐L/G mice (Figure 8G). This was also observed in the livers of patients with ALF (Figure 8H). Under LPS stimulation, primary WT‐ECs secreted significantly more CXCL1 in vitro compared to *Mas1* ^−/−^‐ECs (Figure S10B,C, Supporting Information). CXCL1, which is known to be produced by ECs, plays a crucial role in recruiting neutrophils and promoting NETs formation.^[^ ^27^ ^]^ Next, peripheral blood neutrophils (PBNs) from WT mice were incubated in conditioned medium (CM) from WT‐ or *Mas1* ^−/−^‐ ECs for 4 h. The data showed that WT‐ECs, compared to *Mas1* ^−/−^‐ECs, significantly induced NETs formation in PBNs, as evidenced by higher cfDNA levels and NE/MPO co‐staining (Figure S10D,E, Supporting Information). Finally, pre‐administration of the SHP2 inhibitor SHP099 notably decreased inflammation and cell death in WT‐L/G mice (Figure 8I; Figure S10F, Supporting Information). This activated FXR signaling, inhibiting SphK1 and suppressing the S1P‐driven Raf/MEK/ERK cascade and NETs formation (Figure 8J–L; Figure S10G–I, Supporting Information).

## 3 Discussion

ALF presents a significant global health challenge with high incidence and mortality rates, underscoring the urgency of researching its mechanism is heightened owing to limited treatment options. This study explores the role and molecular mechanism of hepatic Mas signaling in a mouse model of L/G‐induced ALF (Figure S11, Supporting Information). Systemic deficiency of *Mas1* inhibits SHP2, thereby activating FXR signaling. This activation suppressed the S1P‐induced Raf/MER/ERK cascade, NETs formation, and liver inflammation. scRNA‐seq analysis revealed a significant reduction in the number of Cldn1^+^CD177^+^ neutrophils (Neu2) in *Mas1* ^−/−^ mice. This early‐stage neutrophil subcluster is highly associated with NETs formation and is enriched in the pro‐inflammatory TNF signaling pathway. Col4a1^+^ ECs promote the recruitment of Neu2 to the liver and subsequent formation of NETs through Col4a1‐CD44 interactions. These results are vital for understanding inflammation‐related cell damage in the L/G process and developing ALF drugs that counteract Mas signaling.

This study is the first to report that inhibition of Mas receptor provides protection against ALF by reducing NETs formation. Most prior studies,^[^ ^28^ ^]^ including ours on AILI,^[^ ^10^, ^11^ ^]^ has found that Mas receptor antagonism causes inflammation and aggravates cell damage. Two possible reasons could explain the seemingly contradictory protective effects of the Mas blockade following exposure to L/G. First, the different diseases have unique pathogenic mechanisms. In the context of the L/G challenge, NETs‐induced inflammation is a key factor, which is supported by the significant improvement in the WT‐LG mouse phenotype with NETs inhibitors, whereas AILI‐related hepatocyte injury primarily results from drug toxicity rather than damage caused by infiltrating immune inflammatory cells. Second, autophagy is a double‐edged sword that serves as both a protective and harmful cellular adaption mechanism in disease pathogenesis. Current knowledge suggests that NETs formation depends on autophagy,^[^ ^29^ ^]^ highlighting its possible pro‐inflammatory role in ALF. Previous studies, including our AILI study, indicated that Mas activation enhances autophagy for protection.^[^ ^10^, ^30^ ^]^ In contrast, this study demonstrates that Mas antagonism protects against L/G challenge by inhibiting autophagy (data not shown).

The pathogenesis of L/G exposure aims to confine enterogenic endotoxemia effects to the liver in order to mimic human ALF induced by viral hepatitis. In this study, we conducted a comprehensive investigation into various dosing regimens, ultimately determining that a combination of 25 µg kg^−1^ LPS and 250 mg kg^−1^ D‐Gal is optimal. Although this dosage does not induce lethality, it is associated with significant hepatotoxicity, cell death, and inflammatory infiltration. The conclusion regarding the protective effect of antagonistic Mas on ALF is substantiated not only by studies involving *Mas1* gene knockout mice but also through in vivo interventions using Mas agonists and inhibitors in WT‐L/G mice. A779, the first Mas receptor antagonist developed in 1994, offers dose‐dependent protection against L/G exposure for both prevention and treatment. It is worth noting that the impact of A779 and *Mas1* gene knockout on Mas signaling antagonism differed, as evidenced by distinct cell populations in the liver tissue scRNA‐seq data of the WT‐L/G+A779 and *Mas1* ^−/−^‐L/G groups. However, the clinical use of Mas antagonists remains under investigation. Animal studies have indicated potential benefits, such as A779 lowering portal pressure in cirrhotic rats^[^ ^31^ ^]^ and preventing kidney damage in mice fed high‐fat diets.^[^ ^12^ ^]^ Challenges include A779 peptide degradation, necessitating better delivery methods such as nanoparticles, and establishing the best timing, dosage, and combination therapies. Given the impact of the L/G challenge on multiple organs and the widespread expression of Mas across various tissues and cell types, the future application prospects of A779 may surpass those of ALF. However, it is imperative to consider the potential side effects of this drug on other organ systems.

The critical role of enterogenic endotoxemia in ALF prompted investigations into the intestinal alterations. The ABx and co‐housing experiments confirmed that intestinal flora plays a role in the protective effect observed in *Mas1* ^−/−^‐L/G mice. When mice with varying genetic backgrounds were co‐housed, their gut microbiota rapidly aligned producing uniform metabolic byproducts and phenotypic consistency.^[^ ^32^ ^]^ In *Mas1* ^−/‐^ mice, co‐housing with WT mice altered the gut microbiota and metabolism, slightly increasing L/G‐induced gut permeability and liver damage. While separate housing may cause phenotypic differences due to environmental factors, co‐housing reduces these variations In co‐housed WT‐L/G mice, fecal metabolomics revealed a significant decrease in fecal DCA levels, the most altered metabolite, with a notable improvement in the disease phenotype. Notably, L/G exposure significantly increased serum DCA levels in WT mice. In addition, Mas antagonism downregulates DCA concentrations, whereas Mas activation has the opposite effect. Additionally, 16S rDNA sequencing of mouse feces revealed that Eubacterium plexicaudatum, recently described to produce DCA,^[^ ^33^ ^]^ was abundant in WT‐L/G feces (Figure S8E, Supporting Information). DCA, a secondary bile acid, influences FXR signaling both directly and via changes in the gut microbiota. It serves as a weak FXR agonist and may lead to ischemic biliary lesions.^[^ ^34^, ^35^ ^]^ At 100 µm, it inhibits chenodeoxycholic acid (CDCA)‐induced FXR activation^[^ ^36^ ^]^ and acts as an FXR antagonist in colon cancer and metabolic‐associated fatty liver disease.^[^ ^37^, ^38^, ^39^ ^]^ Our research found that *Mas1* ^−/‐^ mice showed a notable decrease in serum and fecal DCA levels along with activated hepatic FXR signaling. Administering DCA to these mice inhibited FXR signaling and worsened liver injury, suggesting that DCA acts as an FXR antagonist in ALF. In summary, effects of DCA vary by concentration and organ: low levels may protect metabolism via FXR activation, while high levels can inhibit FXR and promote disease. Through the in vivo application of the FXR agonist GW4064, we further substantiated that the production of S1P induced by DCA was contingent upon the inhibition of FXR. Our findings establish that FXR serves as a crucial intermediary between Mas and S1P. Furthermore, DCA treatment in *Mas1* ^−/−^‐L/G mice confirmed its harmful effects on L/G processes by promoting S1P‐dependent NETs formation, supporting the findings of a previous renal study.^[^ ^25^ ^]^ Therefore, monitoring serum DCA levels may serve as a valuable tool in tracking the progression of ALF due to its role in the formation of NETs and the subsequent inflammatory response. In addition, this study indicates that SHP2 and DCA independently inhibit FXR through different mechanisms. There is limited research on their direct interaction, except for a study on colon cancer cells, which showed DCA increases SHP2's association with focal adhesion kinase (FAK), hinting at SHP2's role in DCA signaling.^[^ ^40^ ^]^ Further studies using animal models or organoids are required to clarify their interaction with specific diseases.

Currently, the metabolic abnormalities in ALF are poorly understood. The study shows that abnormal sphingoid metabolism in WT‐ECs generates S1P, which activates S1PR2 in neutrophils and promotes NETs formation in ALF mice. This emphasizes the role of the increased Cldn1^+^CD177^+^ neutrophil cluster and its interaction with ECs through the Col4a1‐CD44 pair in S1P‐dependent NETs formation. Additionally, in vitro experiments with primary ECs and PBNs revealed that LPS alters intracellular FXR signaling in WT‐ECs, but not in *Mas1* ^−/−^‐ECs, leading to the release of CXCL1, a neutrophil chemotactic factor that promotes NETs formation. This suggests that the Mas receptor is crucial for controlling the FXR‐S1P production axis in ECs.

A significant limitation of this study was the lack of identification of the specific cell types responsible for initiating and sustaining the Mas signaling in ALF. Preliminary data from our investigation using cell‐specific *Mas1* gene knockout mice shows that the removal of *Mas1* from ECs, myeloid cells, and intestinal epithelial cells significantly improved the ALF disease phenotype. Among these, the L/G phenotypes of intestinal epithelial cell‐specific *Mas1* knockout mice most closely resembled those of *Mas1* ^−/−^‐L/G mice. This suggests a critical role for intestinal epithelial cells in initiating Mas signaling and highlights the importance of studying the intestinal mechanism in regulating this pathway. However, due to space limitations, further details are not provided in this report.

This study confirmed that systemic Mas signaling primarily functions via the SHP2‐FXR‐S1P molecular pathway to regulate NETs formation and influences the inflammatory response during the L/G process. Furthermore, the effective transmission of this signaling axis is contingent on the interaction between ECs and neutrophils, which is mediated by the Col4a1‐Cd44 pair. These findings not only enhance our understanding of the mechanisms underlying ALF but also pave the way for developing targeted therapeutic strategies in clinical practice. Given the diverse array of effector cell sources for Mas signaling, future clinical drug development strategies targeting Mas receptor signal regulation should consider specific cell types.

## 4 Experimental Section

### Human Samples

Liver samples were collected from patients with ALF who underwent liver transplantation (*n* = 4) at Hangzhou First People's Hospital of Westlake University (China), and who underwent liver biopsy (*n* = 2) at Shanghai Tongji Hospital of Tongji University (China); and healthy controls (HC, *n* = 6) who were liver donors. The informed consent was obtained from each subject. The study was carried out under the principles of the Declaration of Helsinki and approved by the research ethics boards of Hangzhou First People's Hospital (ZN‐2024340‐01) and Shanghai Tongji Hospital (K‐2023‐024). Demographic features of enrolled subjects are shown in Supporting Information.

### Experimental Animals

Male WT C57BL/6J mice aged 6–8 weeks were purchased from SLAC Laboratory Animal Company (Shanghai, China). *Mas1* ^−/−^ mice were purchased from Cyagen Biosciences Inc (Suzhou, China). After fasting for 24 h, mice received an intraperitoneal (i.p.) injection of a standard L/G challenge dose (25 µg kg^−1^ LPS and 250 mg kg^−1^ D‐Gal). Samples were collected 5 h post‐injection. To better observe the phenotypic effect, prophylactic ANG‐(1‐7) or a solvent control was administered daily (i.p.) at 2 mg kg^−1^ for 7 days in ALF mice, using 90% of the standard L/G challenge dose. A779 was administered (i.p.) at 1 mg kg^−1^, 2 h prior to L/G challenge. Mice survival was monitored every 2 h for 24 h after administering a lethal dose of L/G challenge (40 µg kg^−1^ LPS and 400 mg kg^−1^ D‐Gal). All mice were maintained in a specific pathogen‐free (SPF) facility. Blood, liver, ileum and feces samples were collected at indicated time points for further analysis. The intervention targets, dosages, and administration modes for the remaining pharmacological compounds used in this study are provided in Supporting Information. The experimental protocols were approved by the Animal Ethics Commission of Shanghai Tongji Hospital, Tongji University School of Medicine (2021‐DW‐007).

### Biochemical and Histological Assessment

Liver injury was assessed through biochemical and histological methods. Serum ALT, TBA, ALP, and SOD levels were measured using commercial kits from Nanjing Jiancheng, China. Liver tissue samples were fixed in 4% paraformaldehyde, dehydrated, embedded, sectioned into 4 µm slices, and stained with hematoxylin and eosin (H&E). TUNEL staining was performed to evaluate cell apoptosis.

### Intrahepatic ECs and Neutrophils Isolation

Under anesthesia, the mouse livers were perfused via the portal vein with Hanks' solution containing protease E (Solarbio, P8360), collagenase IV (Sigma–Aldrich, C5138), and DNase I (Sigma, DN25). After a 10‐min digestion, the livers were removed, homogenized, and filtered through a 70 µm cell filter to create a single‐cell suspension. The cells were purified using a density gradient, discarding the precipitate and keeping the supernatant. This supernatant was then incubated with anti‐CD31 MicroBeads (MiltenyiBiotec #130‐097‐418) to purify ECs. Intrahepatic neutrophils were isolated following the instructions of the mouse tissue neutrophil separation kit (TBD, LZS1100P).

### Peripheral Blood Neutrophils (PBNs) Isolation

Mouse PBNs were isolated using the neutrophil separation reagent kit (Solarbio, P9201) following the manufacturer's instructions.

### Cell Culture and Stimulation

Primary mouse ECs and hepatic neutrophils/PBNs were cultured in endothelial cell medium (ScienCell Research Laboratories #1001) and neutrophil medium (Fuheng, PY‐M107), respectively. ECs were initially maintained in serum‐free medium for a duration of 2 h, followed by a 24‐h incubation with LPS at a concentration of 100 ng mL^−1^. Subsequently, both cells and supernatants were harvested for further analysis. Freshly isolated PBNs were cultured in 24‐well plates that had been pre‐coated with poly‐L‐lysine cover glasses (WHB Scientific, WHB‐24‐CS‐LC) and were treated with ECs‐sourced supernatant (referred to as CM) for 4 h.

### Immunofluorescence (IF)

PBNs were stained with neutrophil elastase (NE, HuaBio, #ET1702‐78), myeloperoxidase (MPO, abcam, ab208670), and DAPI. Images were captured using a fluorescence microscope.

### RNA Sequencing (RNA‐Seq) Analysis

Total RNA was extracted with the Trizol reagent kit (Invitrogen). After strict quality control, RNA samples were prepared for library construction using the Illumina TruseqTM RNA kit and sequenced with Illumina technology. Stringtie calculated FPKM values for each gene or transcript based on Hisat2 alignment results. The analysis includes quality control, annotation from major databases (NR, SwissProt, PFAM, GO, KEGG, STRING), expression quantification, differential analysis, and functional enrichment for known and novel genes/transcripts, using RNA libraries sequenced on the Illumina platform.

### Metabolomics Analysis

Mice liver and feces samples were collected and stored at −80 °C. Prior to LC‐MS analysis, they were thawed on ice and proteins were removed. Using high‐resolution mass spectrometry, non‐targeted metabolomics aims to detect numerous molecular feature peaks in the samples. Metabolomic profiling, encompassing both mass spectrometry and bioinformatics analyses, was conducted in Gene Denovo Biotechnology Co., Ltd. (Guangzhou, China).

### Western Blot Analysis

Proteins from liver and ileum tissues were extracted using RIPA buffer and subsequently quantified using a BCA protein assay kit. Following quantification, 50 µg of protein was resolved on a 10% SDS‐PAGE and transferred onto a PVDF membrane. The membrane was blocked with blocking buffer at room temperature and then incubated overnight at 4 °C with primary antibodies against Cleaved Caspase‐3 (Cl‐cas3; 17, 19 kDa, #9664), Bax (20 kDa, #14796), ERK (42, 44 kDa, #4695), and p‐ERK (42, 44 kDa, #4370), obtained from Cell Signaling Technology (CST, Massachusetts, USA). Antibodies against Bcl‐2 (26 kDa, ab182858) and citrullinated histone H3 (H3cit, 14 kDa, ab281584) were procured from Abcam (MA, USA). Antibodies targeting FXR (70 kDa, A24015) and phosphorylated MEK (p‐MEK, 44 kDa, AP1349) were obtained from Abclonal (Wuhan, China). Antibodies against Raf (73 kDa, #ET1701‐21), MEK (44 kDa, #ET1602‐3), and NE (29 kDa, #ET1702‐78) were sourced from HuaBio (Hangzhou, China). Antibodies for Occludin (59 kDa, T55997), Zo1 (195 kDa, TA5145), and E‐cadherin (120 kDa, TA0131) were acquired from Abmart (Shanghai, China). In this study, antibodies against Mas (42 kDa, NBP1‐78444, Novus), SphK1 (43 kDa, #AP7237C, Abcepta), and S1PR2 (39 kDa, #DF4921, Affinity) were used. Protein levels were normalized using GAPDH (37 kDa, GB11002) or β‐actin (42 kDa, GB15001) antibodies from Servicebio. All imaging was performed using the FluorChem R system (PerkinElmer, Massachusetts, USA) and analyzed with ImageJ software (version 1.8.0, NIH).

### Quantitative Real‐Time PCR Analysis

RNA was extracted from liver and ileum tissues using Trizol (Invitrogen). Reverse transcription and qPCR were performed with Prime‐Script RT and SYBR RT‐PCR kits (Takara) according to the manufacturer's instructions. The mRNA expression levels were quantified using the 2^−ΔΔCT^ method with *Gapdh* or *Actin* as reference genes. Primers were supplied by Sangon Biotech Co., Ltd. (Shanghai, China).

### Enzyme‐Linked Immunosorbent Assay (ELISA)

Serum and plasma samples were prepared and stored at −80 °C. Serum S1P, plasma cfDNA, and supernatant CXCL1 levels were measured using commercial ELISA kits following the manufacturer's instructions.

### Circulating cfDNA Isolation

cfDNA was isolated from 500 µL of plasma utilizing the DNeasy Blood and Tissue Kit (TianGen). In the final step, the DNA was eluted with 30 µL of elution buffer, and its concentration was quantified using a bioanalyzer (Agilent).

### Immunohistochemistry

Immunohistochemical analysis was performed on 4 µm thick formalin‐fixed, paraffin‐embedded mouse liver samples using primary antibodies Mas (1:600), F4/80 (1:100), MPO (1:100), and CD163 (1:500).

### Multiplex Immunohistochemistry (mIHC)

mIHC was conducted on paraffin‐embedded tissue sections using standard primary antibodies sequentially with a TSA 7‐color kit (D110071‐50T, WiSee Bio), followed by DAPI staining. For instance, deparaffinized slides were incubated with anti‐Mas antibody (NBP1‐78444, Novus) for 30 min, then treated with anti‐rabbit HRP‐conjugated secondary antibody (#A10011‐60, Yuanxibio) for 10 min. IF labeling was performed for 10 min using TSA 620 as per the manufacturer's instructions. Slides were washed in TBST buffer, then transferred to 90 °C citrate solution and microwaved at 20% power for 15 min. They were cooled to room temperature in the same solution. Tris buffer washes were done between all steps. The process was repeated sequentially for the following antibodies: anti‐F4/80 (#70076, CST), HNF4α (ab181604, Abcam), Ly6G (#87048, CST), CD31 (#77699, CST), CD45 (ab317446, abcam), Col4a1(AF0510, Affinity), CD44(DF6392, Affinity), Cldn1 (DF6319, Affinity), FXR (ab155124, Abcam), H3Cit (ab281584, Abcam), NE (ET1702‐78, HuaBio), MPO (ab208670, Abcam), SphK1 (AP7237C, abcepta) and SHP2 (AP8471e, abcepta). Each slide received two drops of DAPI, was washed with distilled water, and manually coverslipped. Slides were air‐dried, mounted with anti‐fade medium, and imaged using PANNORAMIC MIDI II. Images were analyzed with Indica Halo software.

### 10x Genomics scRNA‐Seq

Mouse liver tissues were promptly dissociated into single‐cell suspensions, and subsequent data quality assessment and genome alignment were conducted utilizing Cell Ranger (v5.0.0) from 10x Genomics.^[^ ^41^ ^]^ The single‐cell suspension was adjusted to 700–1200 cells µL^−1^, and the 10× Genomics Chromium Next GEM Single Cell 3ʹ Reagent Kits v3.1 (Catalog No. 1000268) were used for machine and library construction as per the manual. The high‐throughput single‐cell transcriptome data were quantified by identifying unique barcode tags for individual cells and UMI tags for different mRNA molecules within each cell. After quality filtering, 25 658 cells were obtained. PCA and UMAP described cell relationships, while Graphcluster and K‐means were used for clustering. Marker gene analysis was performed using the Wilcox rank‐sum test. GO analysis was annotated using NCBI, UniProt, and Gene Ontology. Significant GO categories were identified with Fisher's exact test and p values were corrected using FDR. Monocle 2, CellChatDB, GSEABase (v1.44.0), and SCENIC software were used for pseudotime analysis, cell–cell communication, GSVA, and regulon regulator analysis, respectively.^[^ ^42^, ^43^ ^]^

### ABx Administration

SPF mice were depleted of gut bacteria by administering a cocktail of four antibiotic‐ampicillin, metronidazole, neomycin (each at 1 g L^−1^), and vancomycin (0.5 g L^−1^)‐via gavage for 2 weeks.

### Co‐Housing Experiments

Three‐week‐old *Mas1* ^−/−^ and WT mice were either co‐housed or housed separately for at least 4 weeks. They were kept in independently ventilated cages under SPF conditions, with a 12‐h light cycle, and provided autoclaved food and water.

### Fluorescein Isothiocyanate (FITC)‐Dextran‐Based Permeability Assay

The mice fasted for 4 h, then received 200 mg kg^−1^ of 4 kDa FITC‐dextran via oral gavage. After 90 min, they were sacrificed, and their blood was collected and centrifuged to obtain plasma. The plasma's fluorescence (excitation at 485 nm, emission at 520 nm) was measured with a microplate spectrofluorometer, and FITC‐dextran concentration was determined using a standard curve.

### 16S rDNA Sequencing

Fecal DNA was extracted and amplified using a universal V3V4 16S rDNA primer, a specific barcode, and a fluorescent dye. The PCR products were sequenced on the Illumina NovaSeq 6000 platform, and data extraction was performed using QIIME2 (Version 2022.8). Post quality control, the average sequencing depth for the mice study was 127 737 reads per sample. The DADA2 pipeline processed the 16S rDNA sequencing data, and the relative abundance of bacterial groups was analyzed using qPCR via the delta‐Ct method. Gene Denovo Biotech conducted the 16S rDNA sequencing and analysis.

### Statistical Analysis

Data were shown as mean ± standard deviation (SD) or median ± interquartile range (IQR). Statistical analysis was performed with Prism GraphPad 7.0 and SPSS 22.0. Data normality was checked using histograms and the Shapiro–Wilk test. For two‐group comparisons, normally distributed data were analyzed with a two‐sided Student *t*‐test; otherwise, the Mann–Whitney U test was used. One‐way ANOVA with Tukey's test was used to compare multiple groups. Mouse survival rates were analyzed using the Mantel‐Cox method and Log‐rank test. Sample sizes and *p* values are in the figure legends. *p* < 0.05 was considered statistically significant.

## Conflict of Interest

The authors declare no conflict of interest.

## Author Contributions

B.Y., S.C., X.X., and Z.T. shared co‐first authorship. W.Q., C.Y., and J.L. are co‐senior authors and contributed equally to this work. W.Q., C.Y., J.L., and B.Y. conceived and designed the study. B.Y., S.C., X.X., Z.T., and J.H. acquired the data. B.Y., S.Z., and X.X. analyzed and interpreted the data. S.C., C.L., and L.X. provided administrative, technical, or material support. B.Y., J.L., and C.Y. drafted the article. W.Q., C.Y., and J.L. supervised the study. All authors approved the final version of the manuscript.

## Supporting information



Supporting Information

## Data Availability

The data that support the findings of this study are available from the corresponding author upon reasonable request.
