# Supplementary material for: Mas Signaling Potentiates Neutrophil Extracellular Traps Formation Induced by Endothelial Cells Derived S1P in Mice with Acute Liver Failure
Source: Adv Sci (Weinh). 2025 Apr 26;12(20):2411428. doi: 10.1002/advs.202411428 (PMC12120760; doi:10.1002/advs.202411428)
Supplement: Supplementary file 1 — Supporting Information [file ADVS-12-2411428-s001.docx]

**Mas Signaling Enhances Neutrophil Extracellular Traps Formation Induced by Endothelial Cells-derived S1P in Mice with Acute Liver Failure**

*Bo Yang**^1†^, Shuai Chen**^1†^, Xiaoqi Xia^1†^, Ziwen Tao^1†^, Chun Liu^1^, Shanshan Li**^1^, Shuo Zhang^1^, Jiali Huang**^1^, Lu Xia^1^, Wenqiang Quan^2*^,*

*Changqing Yang^1*^, Jing Li^1*^*

**Table of contents**

Supplementary Methods...................................................................................2

Supplementary Figures and Figure legends.....................................................7

Figure S1..........................................................................................................7

Figure S2..........................................................................................................9

Figure S3........................................................................................................11

Figure S4........................................................................................................12

Figure S5........................................................................................................14

Figure S6........................................................................................................15

Figure S7........................................................................................................17

Figure S8........................................................................................................18

Figure S9........................................................................................................20

Figure S10......................................................................................................21

Figure S11.......................................................................................................23

1. **Supplementary Methods**
   1. **Demographic features of enrolled subjects**

| **Group** | **Specimen Number** | **Gender** | **Age** | **Etiology** |
| --- | --- | --- | --- | --- |
| HC | HC-18 | Female | 59 | / |
| HC | HC-32 | Female | 46 | / |
| HC | HC-39 | Female | 37 | / |
| HC | HC-64 | Male | 28 | / |
| HC | HC-45 | Male | 43 | / |
| HC | HC-76 | Female | 60 | / |
| ALF | ALF-605691039 | Female | 67 | HBV |
| ALF | ALF-607929801 | Male | 54 | HBV |
| ALF | ALF-604529567 | Female | 62 | HBV |
| ALF | ALF-603127832 | Female | 60 | HBV |
| ALF | ALF-608769828 | Male | 58 | HBV |
| ALF | ALF-609867823 | Female | 53 | HBV |

- 1. **Detailed information of *in vivo* drug administrations.**

| **Name** | **Cat. No.** | **Function** | **Dose and**  **frequency** | **Time point** | **Administration**  **mode** |
| --- | --- | --- | --- | --- | --- |
| LPS | Sigma-  Aldrich  SMB00704 | ALF modeling | 25 µg kg^-1^  One time | 5 h | Phosphate-buffered solution (PBS)  intraperitoneal (i.p.) |
| D-Gal | Sigma-  Aldrich  34539-M | ALF modeling | 250 mg kg^-1^  One time | 5 h | PBS |
| ANG-(1-7) | MCE  HY-12403 | Mas receptor agonist | 2 mg kg^-1^  1 w continuously | 1 w before L/G | Water  i.p. |
| A779 | MCE  HY-P0216 | Mas receptor inhibitor | 1 mg kg^-1^  One time | 2 h before L/G | Water  i.p. |
| GSK484 | MCE  HY-100514 | PAD4 inhibitor | 4 mg kg^-1^  1 w continuously | 1 w before L/G | DMSO  i.p. |
| (Z)-Guggulsterone | MCE  HY-110066 | FXR antagonist | 40 mg kg^-1^  1 w continuously | 1 w before L/G | DMSO  gavage |
| CD44mAb | 103002 | Purified anti-mouse CD44 Antibody | 100 µg per mouse the first day then  50 µg per mouse  6 d continuously | 6 d before L/G | PBS  i.p. |
| PF543 | Selleck  S7177 | SphK1 inhibitor | 10 mg kg^-1^  Two time | 2 d before L/G | Water  i.p. |
| SHP099 | Selleck  S8278 | SHP2 inhibitor | 15 mg kg^-1^  Three time | 3 d before L/G | Water  i.p. |
| Ampicillin | Sigma-  Aldrich  A5354 | Destruction of gut microbiome | 1 g L^-1^  2 w continuously | 2 w before L/G | DMSO  gavage |
| Metronidazole | Sigma-  Aldrich  M1547 | Destruction of gut microbiome | 1 g L^-1^  2 w continuously | 2 w before L/G | acetic acid  gavage |
| Neomycin | Sigma Aldrich  N1142 | Destruction of gut microbiome | 1 g L^-1^  2 w continuously | 2 w before L/G | 0.9% NaCl  gavage |
| Vancomycin | Sigma Aldrich  V2002 | Destruction of gut microbiome | 0.5 g L^-1^  2 w continuously | 2 w before L/G | DMSO  gavage |
| Deoxycholic acid | Sigma Aldrich  D2510 | FXR inhibitor | 100 mg kg^-1^  1 w continuously | 1 w before L/G | DMSO  gavage |
| GW4064 | MCE  HY-50108 | FXR agonist | 30 mg kg^-1^  1 w continuously | 1 w before L/G | DMSO  gavage |
| Fluorescein isothiocyanate (FITC) | Sigma-  Aldrich  68059 | FITC-dextran-based permeability assay | 200 mg kg^-1^  One time | 90 min | Water  gavage |

- 1. **Primary antibodies and ELISA kits.**

| **Name** | **Citation** | **Supplier** | **Cat no.** | **Clone no.** |
| --- | --- | --- | --- | --- |
| Cleaved Caspase-3 (Asp175) | PMID:34772930 | CST | 9664 | 5A1E |
| Bax | PMID:34601488 | CST | 14796 | D3R2M |
| LC3B | PMID:34435379 | CST | 3868 | D11 |
| FXR | PMID:37905695 | abcam | ab155124 | polyclonal |
| Histone H3 (citrulline R2 + R8 + R17) | PMID:37424151 | abcam | Ab281584 | RM1001 |
| Bcl-2 | PMID:33156578 | abcam | ab182858 | EPR17509 |
| Neutrophil Elastase (NE) | PMID: 36890868 | HuaBio | ET1702-78 | JF098-6 |
| P62 | PMID:32858179 | abcam | ab109012 | EPR4844 |
| ERK1/2 | PMID: 38764835 | HuaBio | ET1601-29 | SA43-03 |
| Phospho-ERK1 (T202 + Y204) + ERK2 (T185 + Y187) | PMID: 38764835 | HuaBio | ET1610-13 | SC58-01 |
| Phospho-MEK1/2 (S218 + S222) | PMID: 38427603 | HuaBio | ET1609-50 | ST0490 |
| GAPDH | PMID：33846290 | Servicebio | GB11002 | Polyclonal |
| F4/80 | PMID:33432198 | Servicebio | GB11027 | Polyclonal |
| MPO | PMID:33417512 | abcam | ab208670 | EPR20257 |
| MEK1/2 | PMID: 39002703 | HuaBio | ET1602-3 | SR13-07 |
| F4/80 | PMID:31594939 | Invitrogen | MF48020 | BM8 |
| Ly6G | PMID:23273920 | Invitrogen | 12-5931-82 | RB6-8C5 |
| GAPDH | PMID: 35259473 | absin | abs132004 | Polyclonal |
| Mas | PMID: 34529881 | Novus | NBP1-78444 | Polyclonal |
| CD68 | PMID: 16888915 | Biolynx | BX50031 | BP6036 |
| Raf1 | PMID: 32416844 | HuaBio | ET1701-21 | JJ086-06 |
| SphK1 | PMID: 32522601 | abcepta | AP7237C | Polyclonal |
| HNF4α | PMID: 33854885 | abcam | ab181604 | EPR16885 |
| CD31 | PMID: 35393952 | CST | 77699S | D8V9E |
| GFAP | PMID: 34174183 | abcam | ab68428 | EPR1034Y |
| β-actin | PMID: 38184594’ | abcepta | AM1021b | Monoclonal |
| SHP2 | PMID: 24173294 | abcepta | AP8471e | Polyclonal |
| EDG5 |  | Affinity | DF4921 | Polyclonal |
| F4/80 | PMID: 35393952 | CST | 70076 | D2S9R |
| Ly-6G | PMID: 34899755 | CST | 87048 | E6Z1T |
| CD44 | PMID: 37500654 | Affinity | DF6392 | Polyclonal |
| Collagen IV | PMID: 31220559 | Affinity | AF0510 | Polyclonal |
| Claudin 1 | PMID: 36608780 | Affinity | DF6919 | Polyclonal |
| Occludin | PMID: 37521471 | Abmart | TD7504 | Polyclonal |
| Zo1 | PMID: 38761152 | Abmart | TA5145 | Polyclonal |
| E-cadherin | PMID: 37185865 | Abmart | TA0131 | Polyclonal |
| ALT | / | JianCheng | C009-2-1 | / |
| TBA | / | JianCheng | E003-2-1 | / |
| ALP | / | JianCheng | A059-2-2 | / |
| SOD | / | JianCheng | A001-3-2 | / |
| S1P | / | MeiMian | MM-44778M1 | / |
| DCA | / | MeiMian | MM-44754M1 | / |
| cfDNA | / | TianGen | DP304 | / |
| CXCL1 | / | MeiMian | MM-43835M2 | / |

- 1. **Organisms**

| **Name** | **Citation** | **Supplier** | **Strain** | **Sex** | **Age** |
| --- | --- | --- | --- | --- | --- |
| C57BL/6J |  | SLAC Laboratory Animal Company (Shanghai, China) | C57BL/6J | Male | 6-8 weeks |
| C57BL/6N-  *Mas1*^em1cyagen^ | https://www.cyagen.com/cn/zh-cn/sperm-bank-live/17171 | Cyagen Biosciences Inc (Suzhou, China) | KOCMP-17171-Mas1-B6N-VA | Male | 6-8 weeks |

- 1. **Sequence based reagents**

| **Name** | **Sequence** | **Supplier** |
| --- | --- | --- |
| RT-PCR primer  mouse *Mas1* | F AGAAATCCCTTCACGGTCTACA  R GTCACCGATAATGTCACGATTGT | Sangon Biotech, Shanghai, China |
| RT-PCR primer  mouse *Mafg* | F GACCCCCAATAAAGGAAACAA  R TCAACTCTCGCACCGACAT | Sangon Biotech, Shanghai, China |
| RT-PCR primer  mouse *Nr1h4* | F GACGTGGGTCACTTTGGACT  R ACATCCCCATCTCTCTGCAC | Sangon Biotech, Shanghai, China |
| RT-PCR primer  mouse *Nr0b2* | F TCTGCAGGTCGTCCGACTAT  R CAGGCAGTGGCTGTGAGAT | Sangon Biotech, Shanghai, China |
| RT-PCR primer  mouse *Ptpn11* | F TCCATGGTCACTTGTCTGGA  R GACGTGGGTCACTTTGGACT | Sangon Biotech, Shanghai, China |
| RT-PCR primer  mouse *S1pr2* | F ATGGGCGGCTTATACTCAGAGF  R GCGCAGCACAAGATGATGAT | Sangon Biotech, Shanghai, China |
| RT-PCR primer  mouse *Slc51b* | F GATGCGGCTCCTTGGAATTA  R GGAGGAACATGCTTGTCATGAC | Sangon Biotech, Shanghai, China |
| RT-PCR primer  mouse *Sphk1* | F GCCACCTCCAGAAGAACC  R ACTTTAGAAATAACCTCCCATA | Sangon Biotech, Shanghai, China |
| RT-PCR primer  mouse *Cldn1* | F CTGGAAGATGATGAGGTGCAGAAGA  R CCACTAATGTCGCCAGACCTGAA | Sangon Biotech, Shanghai, China |
| RT-PCR primer  mouse *Jam* | F GAAGGATACGTGCAAGGGAGATT  R CAAGTGCCAGACCCCATTGT | Sangon Biotech, Shanghai, China |
| RT-PCR primer  mouse *Tjp3* | F TCGGCATAGCTGTCTCTGGA  R GTTGGCTGTTTTGGTGCAGG | Sangon Biotech, Shanghai, China |
| RT-PCR primer  mouse *Ocln* | F ATGTCCGGCCGATGCTCTC  R TTTGGCTGCTCTTGGGTCTGTAT | Sangon Biotech, Shanghai, China |
| RT-PCR primer  mouse *β-actin* | F GGCTGTATTCCCCTCCATCG  R CCAGTTGGTAACAATGCCATGT | Sangon Biotech, Shanghai, China |
| RT-PCR primer  mouse *Gapdh* | F AGGTCGGTGTGAACGGATTTG  R GGGGTCGTTGATGGCAACA | Sangon Biotech, Shanghai, China |

- 1. **Deposited data**

| **Name of repository** | **Identifier** | **Sample** | **Group** |
| --- | --- | --- | --- |
| RNA sequencing  (RNA-seq) | OE Biotech | Mouse liver | WT-L/G vs. *Mas1*^-/-^-L/G (n=3 per group) |
| Single-cell RNA  sequencing (scRNA-seq) | OE Biotech | Mouse liver | WT, WT-L/G, WT-L/G+A779, *Mas1*^-/-^-L/G  (n=1 per group, mixed sample collection) |
| Metabolomics | Gene Denovo Biotech | (1) Mouse liver  (2) Mouse feces | (1) WT-L/G, *Mas1*^-/-^-L/G (n=6 per group)   1. WT-L/G, WT-L/G(Co), *Mas1*^-/-^-L/G, *Mas1*^-/-^-L/G (Co) (n=6 per group) |
| 16S rDNA | Gene Denovo Biotech | Mouse feces | WT-L/G, WT-L/G(Co), *Mas1*^-/-^-L/G, *Mas1*^-/-^-L/G (Co) (n=6 per group) |

- 1. **Software**

| **Software name** | **Manufacturer** | **Version** |
| --- | --- | --- |
| Graphpad Prism | GraphPad Software | 7.0 |
| Image J | NIH Image for the macintosh | 1.8.0 |
| SPSS | IBM | 22.0 |

**2. Supplementary Figures and Legends**

**
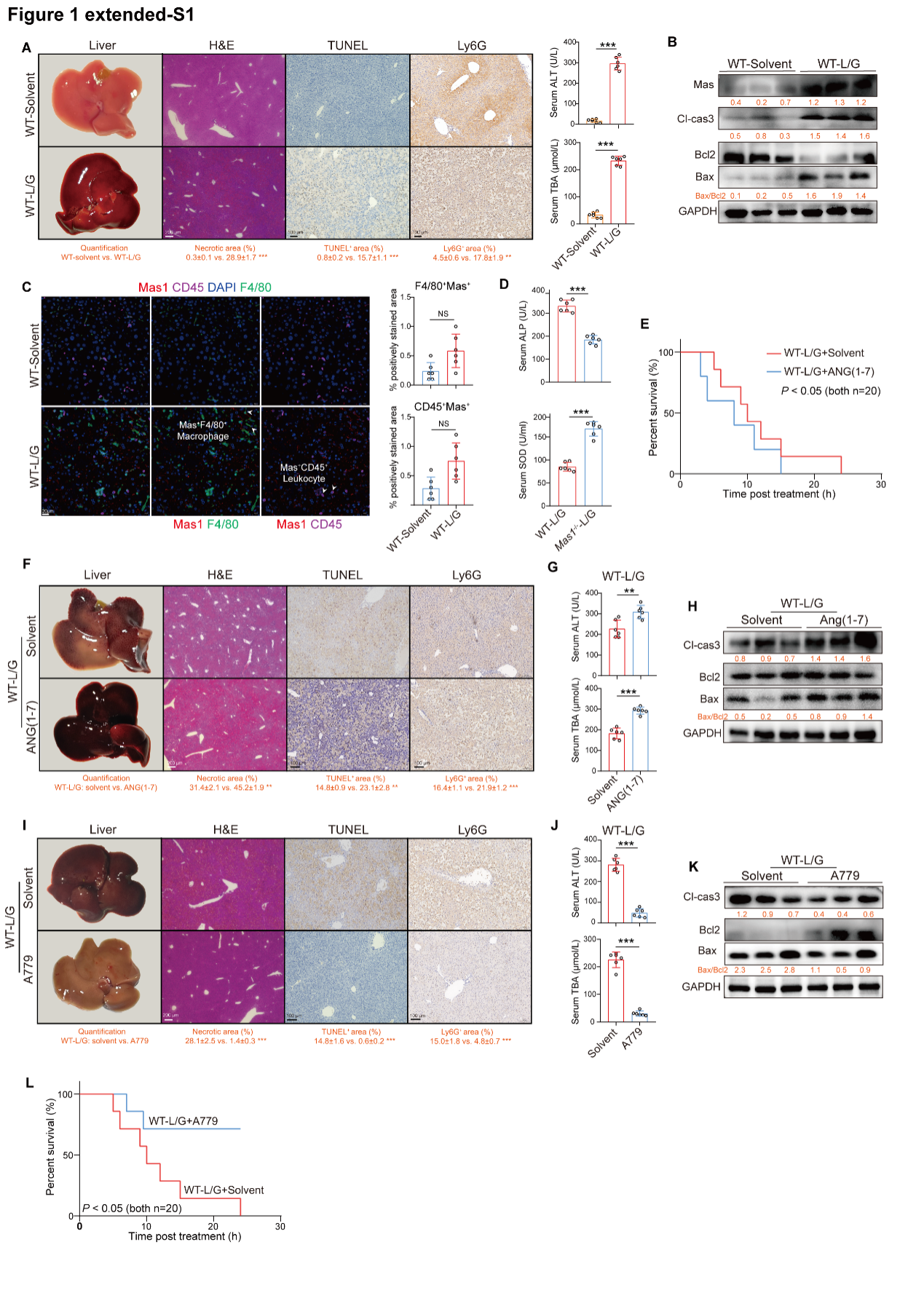
**

**Figure S1. Related to Figure 1.** Systemic *Mas1* deficiency protects mice from L/G challenge. A) Representative liver photographs and immunohistochemical staining with the quantification (below) of H&E, TUNEL and Ly6G (two-sided Student’s t-test, p = 6.32 × 10^−4^, p = 3.93 × 10^−4^ and p = 2.67 × 10^−3^ from left to right). Serum levels of ALT and TBA (two-sided Student’s t-test, p = 6.31 × 10^−4^ and p = 5.47 × 10^−4^ from top to bottom). Scale bars are shown as indicated. B) Representative liver immunoblots of Mas and markers for cell death (cleaved-caspase3, Bax, Bcl2) with the quantification (below). C) Representative mIHC staining of Mas^+^ macrophages (F4/80) and leukocytes (CD45) (p = 0.42 and 0.59 from top to bottom). Scale bars are shown as indicated. D) Serum levels of ALP and SOD in WT and *Mas1*^-/-^ mice challenged with a standard dose of L/G or solvent control for 5 hours (n = 6 per group) (two-sided Mann-Whitney U test, p = 2.91 × 10^−4^ and p = 3.55 × 10^−4^ from top to bottom). WT-L/G mice were prophylactically treated with ANG-(1-7) or solvent control (n = 6 per group, E-H). E) Survival curves of mice challenged with a lethal dose of L/G (both n = 20, Log-rank test, p = 1.90 × 10^−2^). F) Representative liver photographs and immunohistochemical staining with the quantification of H&E, TUNEL and Ly6G (two-sided Student’s t-test, p = 3.19 × 10^−3^, p = 4.87 × 10^−3^ and p = 5.92 × 10^−4^ from left to right). Scale bars are shown as indicated. G) Serum levels of ALT and TBA (two-sided Student’s t-test, p = 5.89 × 10^−3^ and p = 3.81 × 10^−4^ from top to bottom). H)Representative liver immunoblots of markers for cell death (cleaved-caspase3, Bax, Bcl2) with the quantification (below). WT mice were treated with A779 or solvent control before L/G challenge (n = 6 per group, I-L). I) Representative liver photographs and immunohistochemical staining with the quantification of H&E, TUNEL and Ly6G (two-sided Student’s t-test, p = 5.21 × 10^−4^, p = 3.97 × 10^−4^ and p = 5.04 × 10^−4^ from left to right). Scale bars are shown as indicated. J) Serum levels of ALT and TBA (two-sided Student’s t-test, p = 8.01 × 10^−4^ and p = 6.58 × 10^−4^ from top to bottom). K) Representative liver immunoblots with the quantification (below). L) Survival curves of mice challenged with a lethal dose of L/G (both n = 20, Log-rank test, p = 2.31 × 10^−2^).Data are presented as mean ± SD or median ± IQR (***p* < 0.01, ****p* < 0.001).


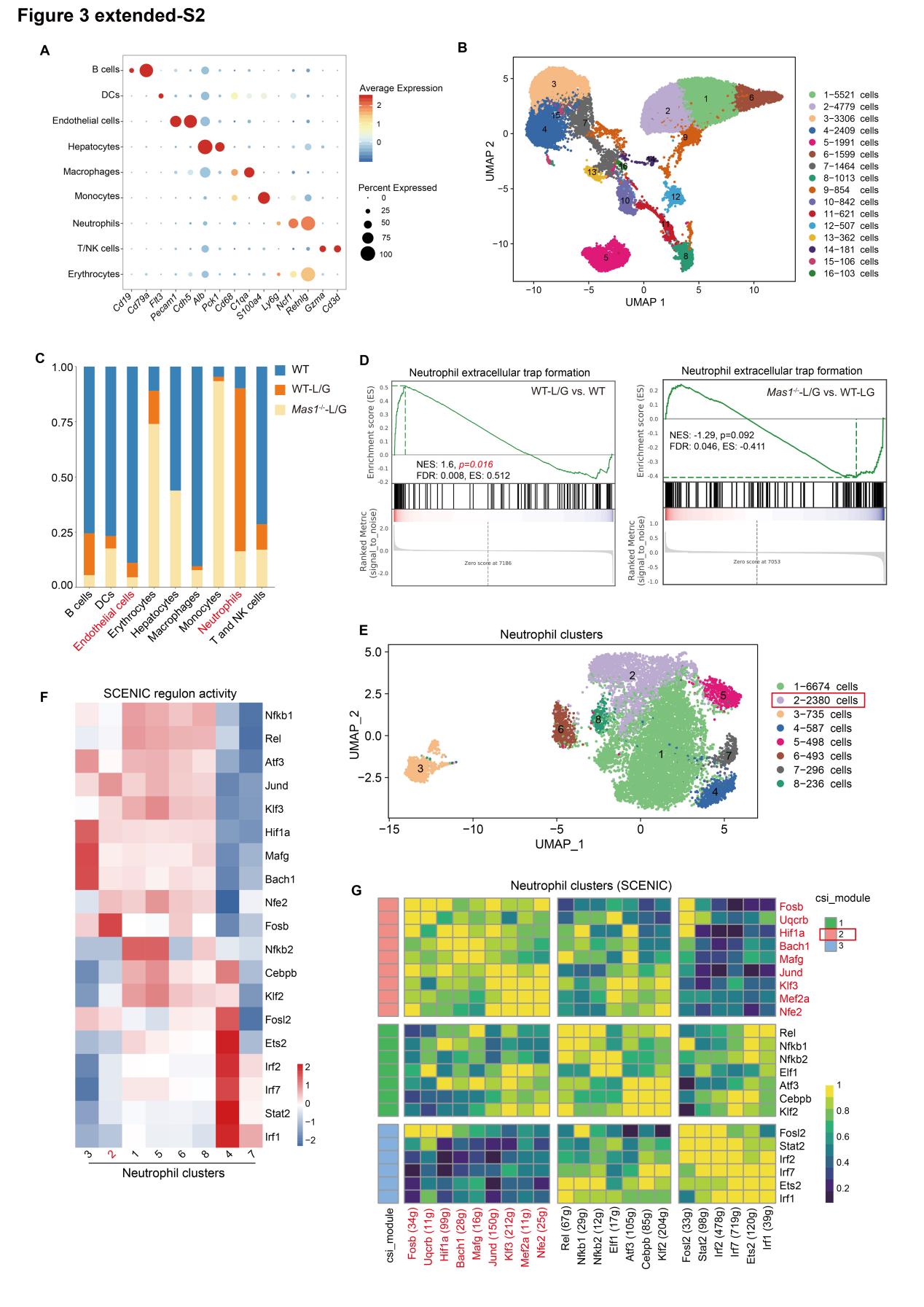


**Figure S2. Related to Figure 3.** Single-cell annotation of Cldn1^+^CD177^+^ neutrophils in *Mas1*^-/-^-L/G mice. A) Dot plots illustrating scaled expression of the gene signature for different cell types. B) The UMAP plot of 25, 658 single cells from liver samples. C) Percentage of different cell types in WT, WT-L/G and *Mas1*^-/-^-L/G mouse liver samples. D) Single-cell GSEA of KEGG enrichment plot in neutrophils (left, WT-L/G vs. WT; right, *Mas1*^-/-^-L/G vs WT-L/G). E) The UMAP plot of 11, 899 single cells from neutrophils. F) Heatmap of single-cell regulatory network inference and clustering (SCENIC) regulons activity in neutrophil clusters. G) Heatmap of regulons connection specificity index in neutrophil clusters.

**
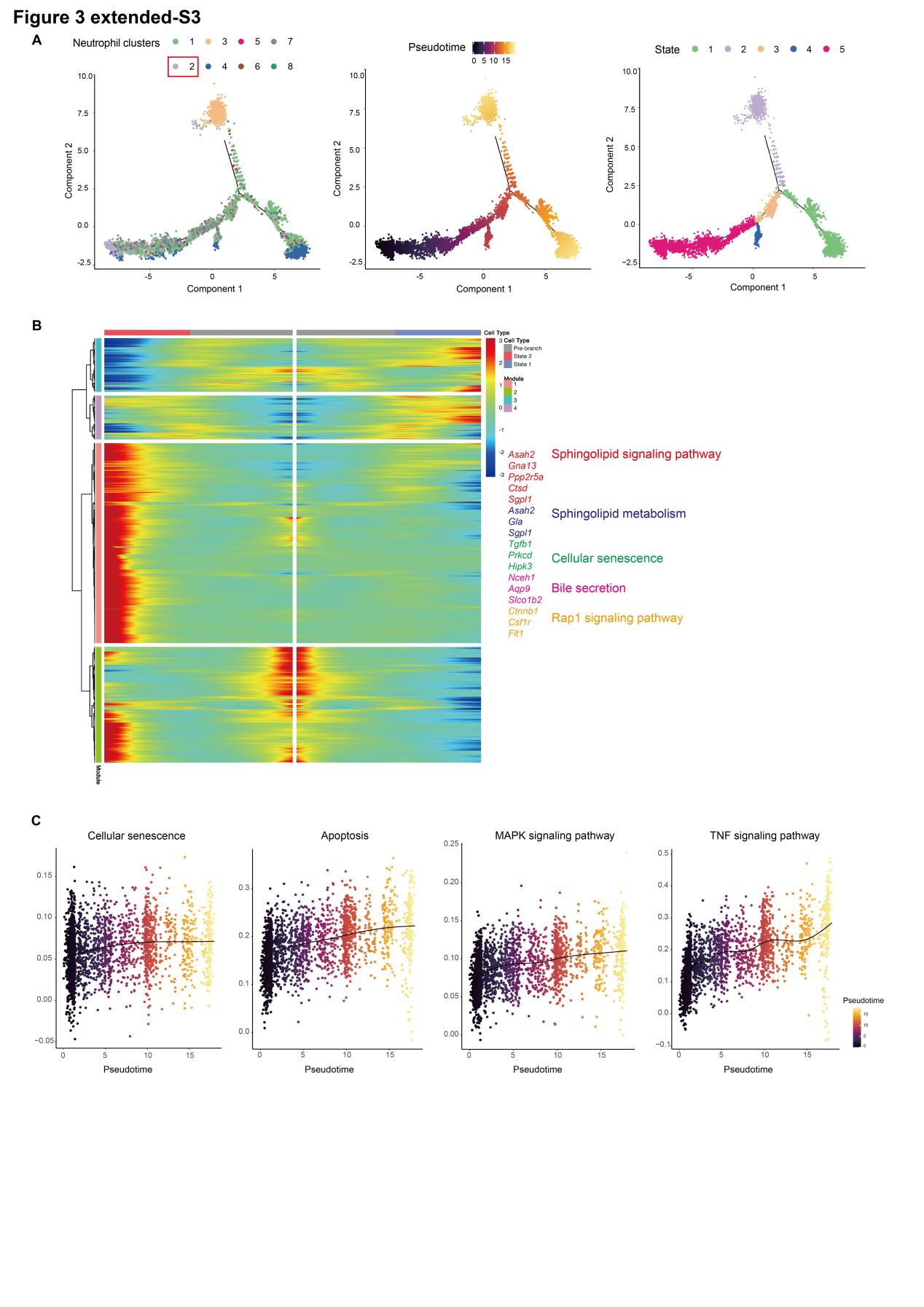
**

**Figure S3. Related to Figure 3.** Single-cell annotation of Cldn1^+^CD177^+^ neutrophils in *Mas1*^-/-^-L/G mice. A) Monocle analyses show the development of neutrophils clusters. B) Heatmap illustrates expression of representative identified genes by neutrophils clusters enriched in the indicated pathways. C) Smoothed expression curves of representative pathways over pseudotime.

**
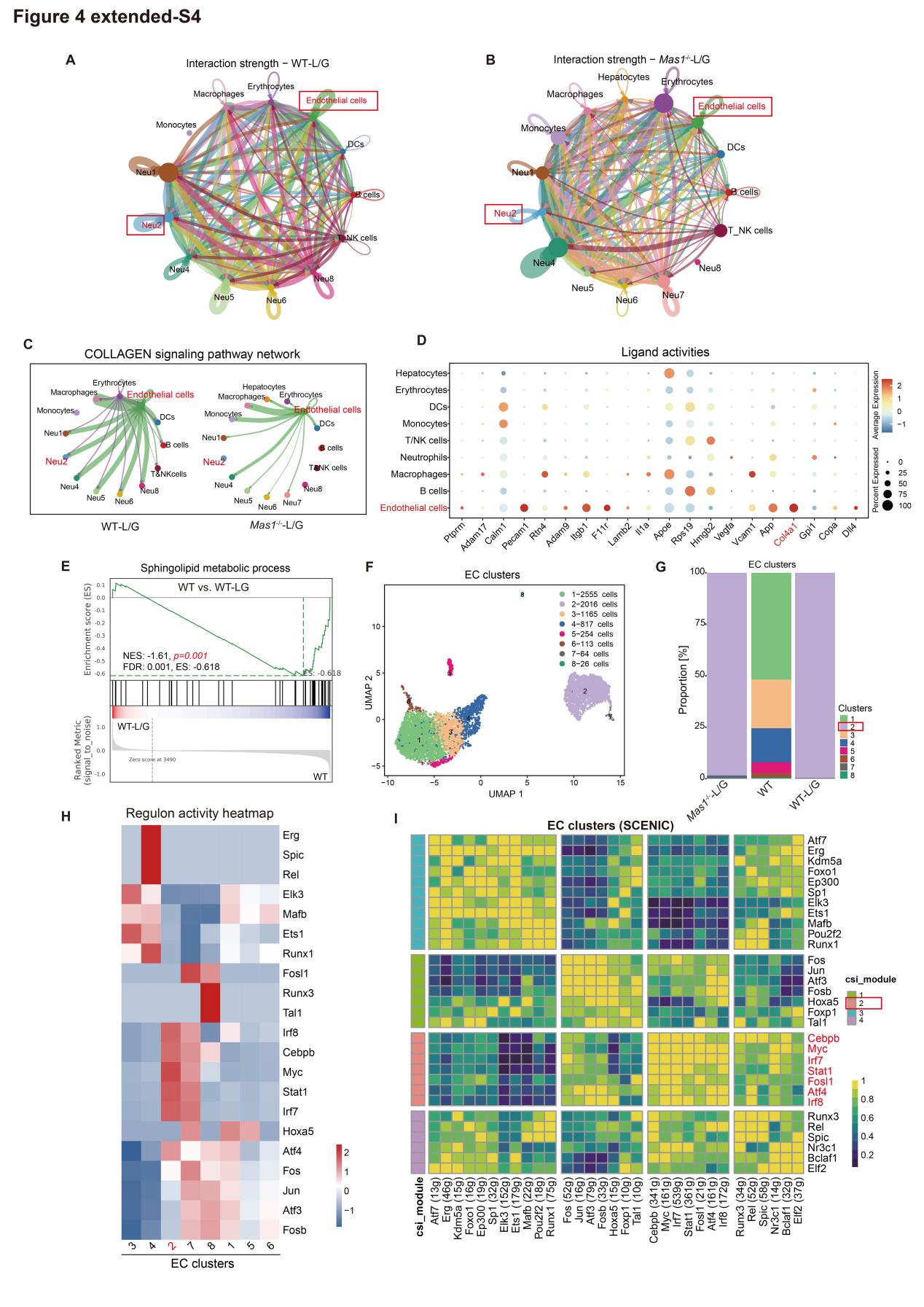
**

**Figure S4.** **Related to Figure 4.** Col4a1^+^ ECs trigger NETs formation in Cldn1^+^CD177^+^ neutrophils via Col4a1-CD44 interaction. A, B) Circle plots illustrate ligand-receptor interaction strength between neutrophil clusters and other cell types in WT-LG (A) and *Mas1*^-/-^-LG (B) mouse livers, with line thickness indicating the interaction strength. C) Network of neutrophil clusters and other liver cell types in the collagen signaling pathway. D) Dot plots show ligand activities of different liver cell types. E) GSEA of KEGG enrichment plot in ECs (scRNA-seq). F) The UMAP plot of 7, 010 single cells from ECs. G) Histogram shows the proportion of EC clusters. H) Heatmap of SCENIC regulons activity in EC clusters. I) Heatmap of SCENIC regulons connection specificity index in EC clusters.


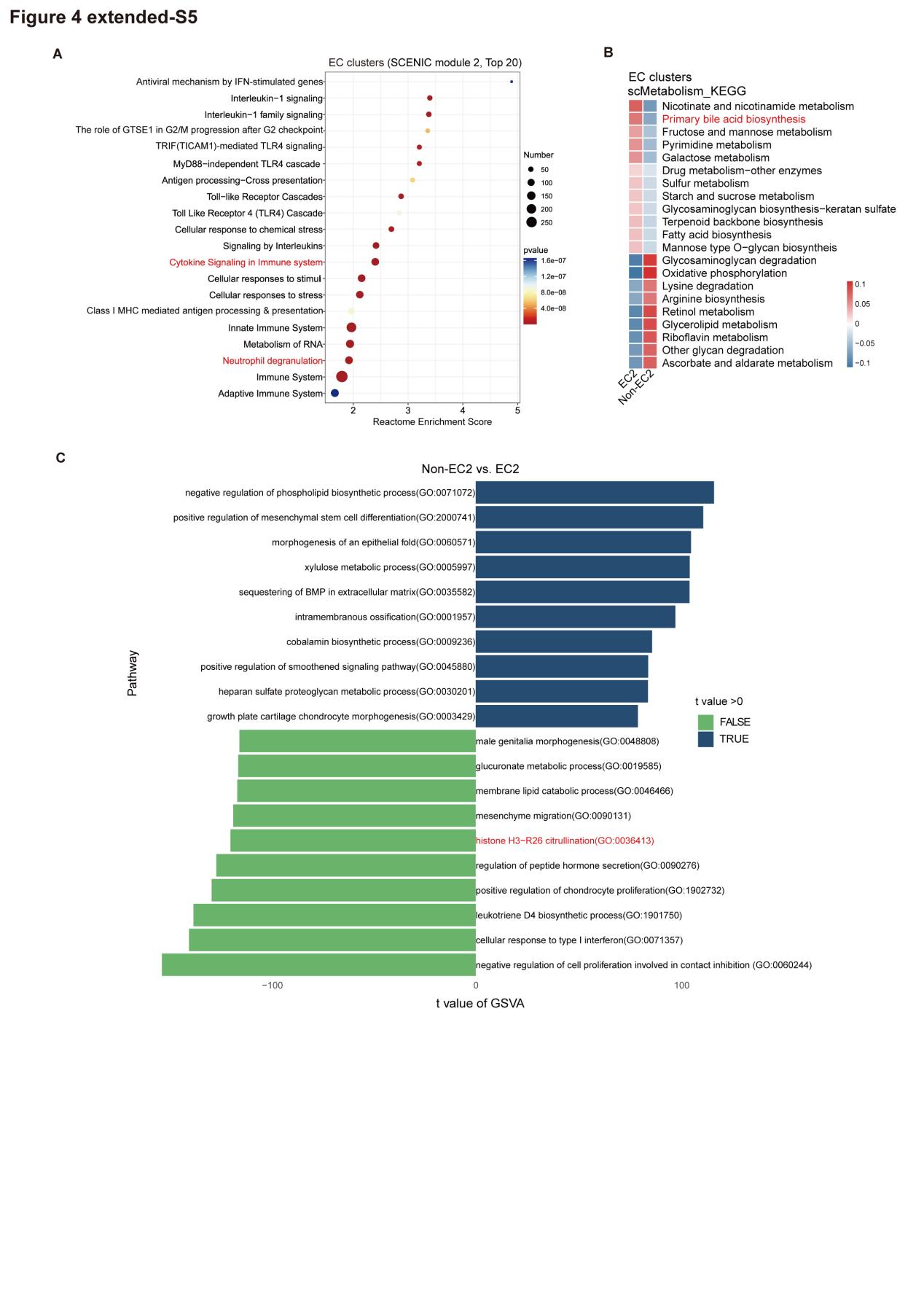


**Figure S5. Related to Figure 4.** Col4a1^+^ ECs trigger NETs formation in Cldn1^+^CD177^+^ neutrophils via Col4a1-CD44 interaction. A) Top 20 Reactome enrichment of Module 2 target genes identified by SCENIC analysis of EC2. B) Heatmap of scMetabolism KEGG enrichment of EC2 and non-EC2. C) Barplot of Gene Set Variation Analysis (GSVA) score of non-EC2 vs. EC2 (t value＞0: upregulated, t value＜0: downregulated).


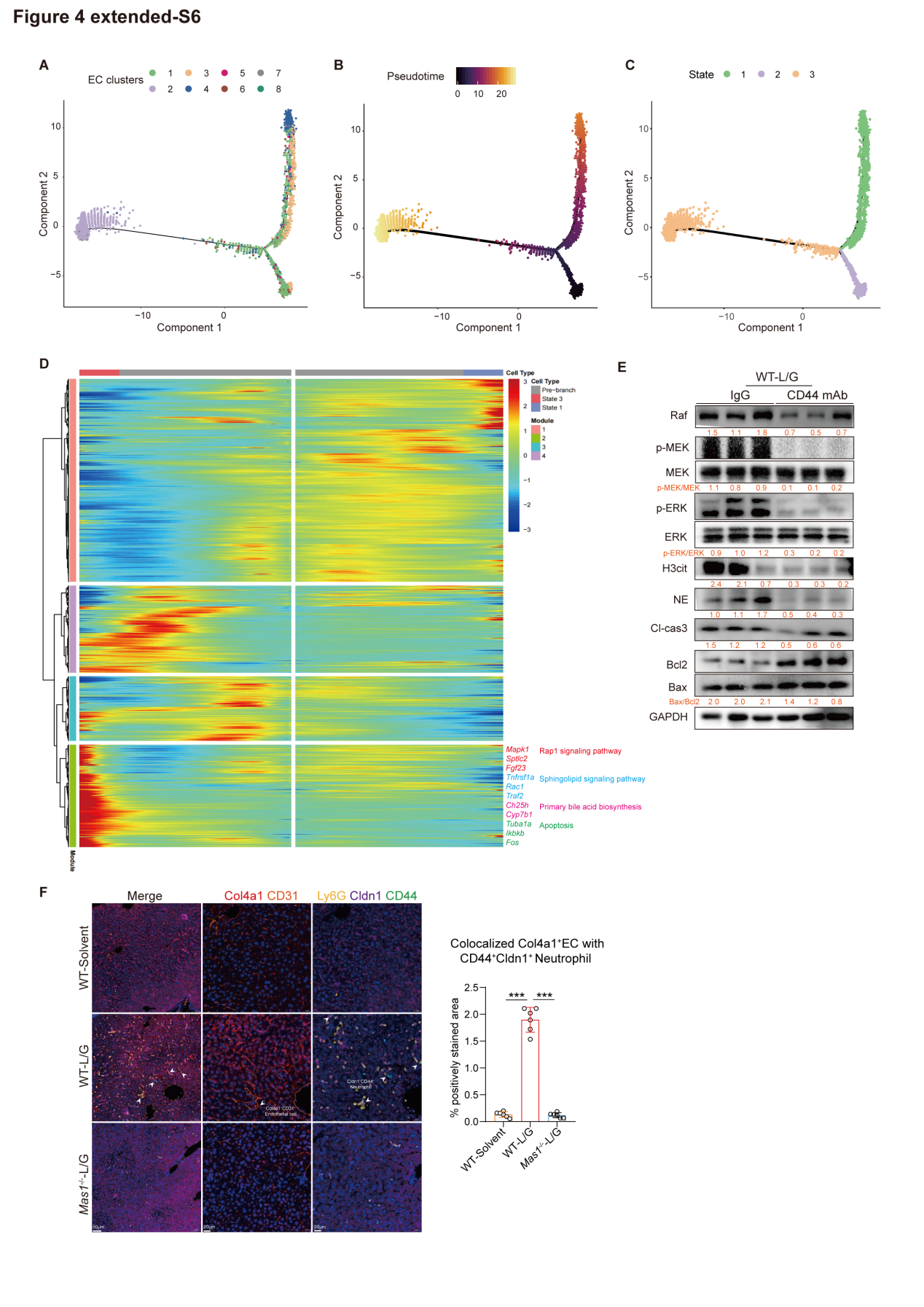


**Figure S6. Related to Figure 4.** Col4a1^+^ ECs trigger NETs formation in Cldn1^+^CD177^+^ neutrophils via Col4a1-CD44 interaction. (A-C) Monocle analyses show the development of EC clusters over pseudotime. D) Heatmap illustrates expression of representative identified genes by EC clusters enriched in the indicated pathways. E) WT-L/G mice were pre-treated with CD44 mAb or IgG control (n = 6 per group). Representative liver immunoblots with the quantification (below). F) Representative mIHC staining (left) and the quantification (right) of co-localization between intrahepatic Col4a1^+^ ECs and Cldn1^+^CD44^+^ neutrophils (One-way ANOVA with Tukey’s test, two-sided Student’s t-test, p = 4.92 × 10^−4^ and p = 3.75 × 10^−4^ from left to right). Scale bars are shown as indicated. Data are presented as mean ± SD (****p* < 0.001).


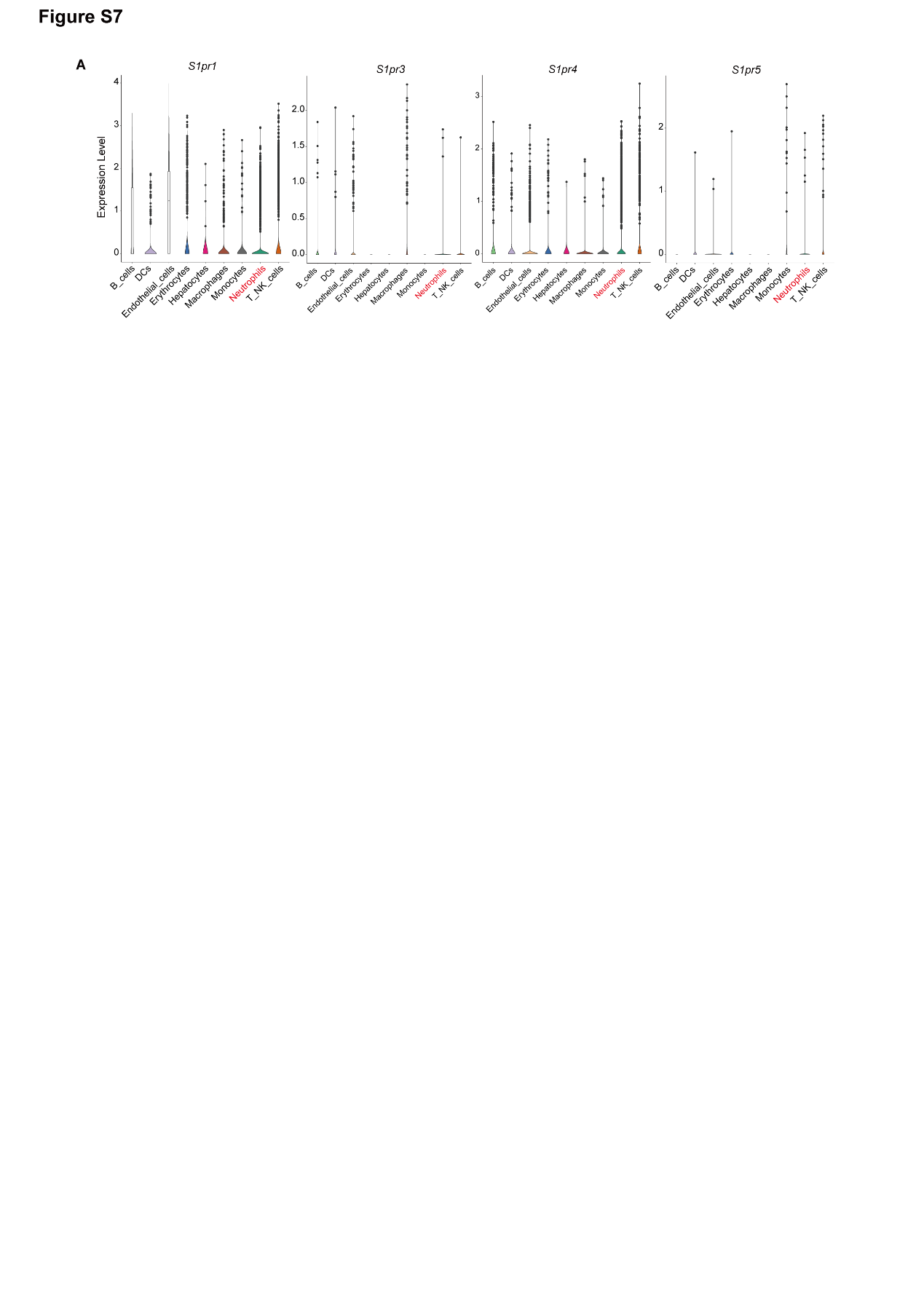


**Figure S7. Related to Figure 5.** Systemic *Mas1* deficiency inhibits S1P-induced NETs formation. (A) Violin boxplot illustrates the expression levels of *S1pr1*, *S1pr3*, *S1pr4*, and *S1pr5* across different cell types.

**
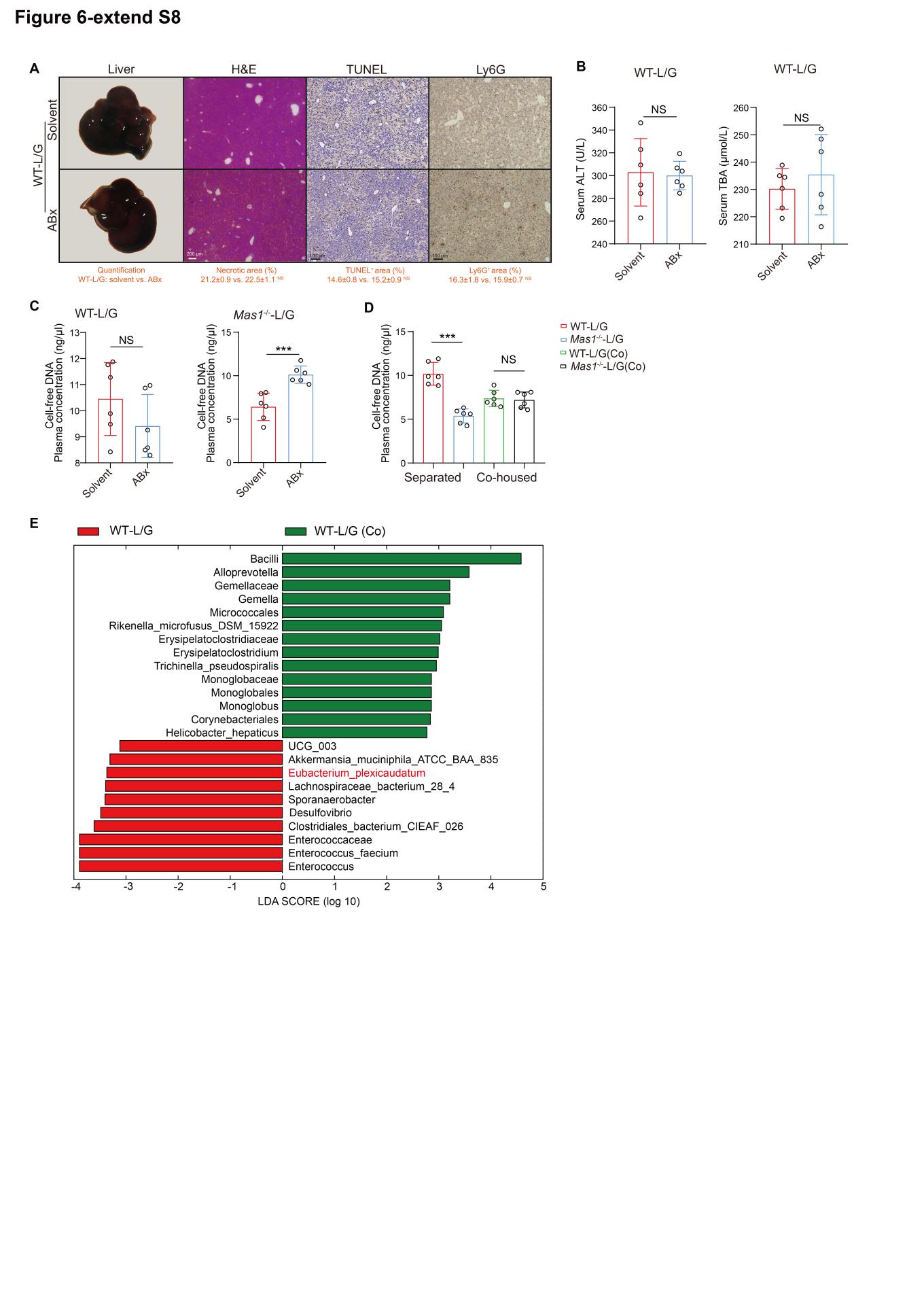
**

**Figure S8. Related to Figure 6.** Systemic Mas modulates gut microbiota and DCA production in mice after L/G challenge. WT-L/G and *Mas1*^-/-^-L/G mice were prophylactically treated with ABx or solvent control (n = 6 per group, A-C). A) Representative liver photographs and immunohistochemical staining with the quantification (below) of H&E , TUNEL and Ly6G (two-sided Student’s t-test, p = 0.68, p = 0.59 and p = 0.89 from left to right). Scale bars are shown as indicated. B) Serum levels of ALT and TBA (two-sided Student’s t-test, p = 0.98 and p = 0.74 from left to right). C) Plasma levels of cell-free DNA (two-sided Student’s t-test, p = 0.83 and p = 3.19 × 10^−4^ from left to right). *Mas1*^-/-^ and WT mice were co-housed or separated for 4 weeks before L/G challenge. D) Plasma levels of cell-free DNA (n = 6 per group; One-way ANOVA with Tukey’s test, two-sided Student’s t-test, p = 5.62 × 10^−4^, p = 0.87 from left to right). E) LEfSe (Linear discriminant analysis effect size) showing the LDA sores of mice feces from WT-L/G (Co) and WT-L/G groups. Data are presented as mean ± SD (****p* < 0.001; NS, not significant).

**
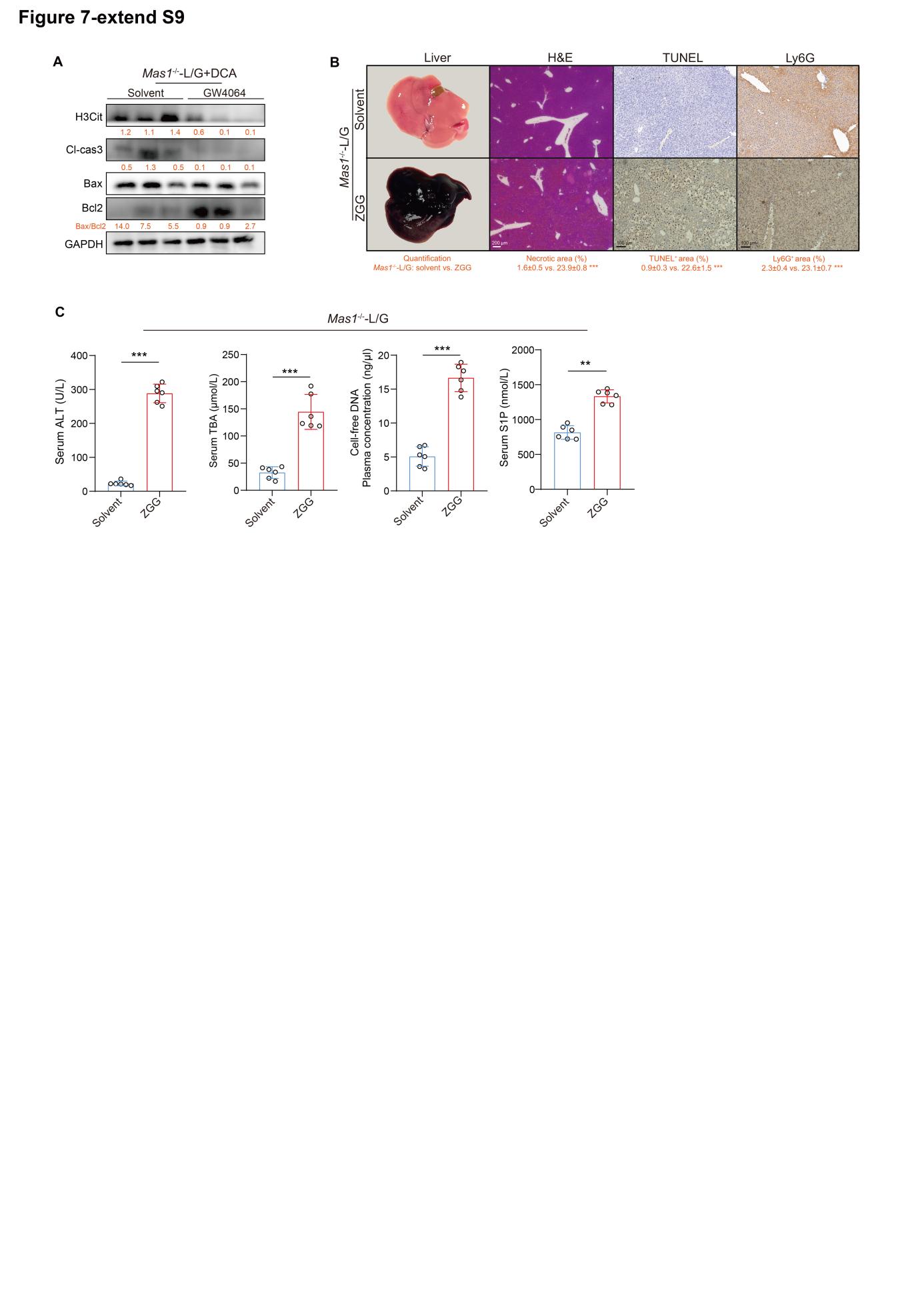
**

**Figure S9. Related to Figure 7.** FXR serves as the pivotal upstream regulator of S1P in L/G challenge. Mice were pre-treated with oral FXR agonist GW4064 or solvent control (n = 6 per group) (A). A) Representative immunoblots of liver tissues with the quantification (below). *Mas1*^-/-^-L/G mice were pre-treated with oral FXR antagonist (Z)-Guggulsterone (ZGG), or solvent control (n = 6 per group, B-C). B) Representative liver photographs and immunohistochemical staining with quantification of H&E, TUNEL and Ly6G (two-sided Student’s t-test, p = 6.39 × 10^−4^ and p = 3.22 × 10^−4^, p = 8.92 × 10^−4^ from left to right). Scale bars are shown as indicated. C) Serum levels of ALT and TBA (two-sided Student’s t-test, p = 7.63 × 10^−4^ and p = 8.49 × 10^−4^ from left to right). Plasma levels of cell-free DNA (two-sided Student’s t-test, p = 6.54 × 10^−4^). Serum levels of S1P (two-sided Student’s t-test, p = 7.29 × 10^−3^). Data are presented as mean ± SD (***p* < 0.01, ****p* < 0.001).
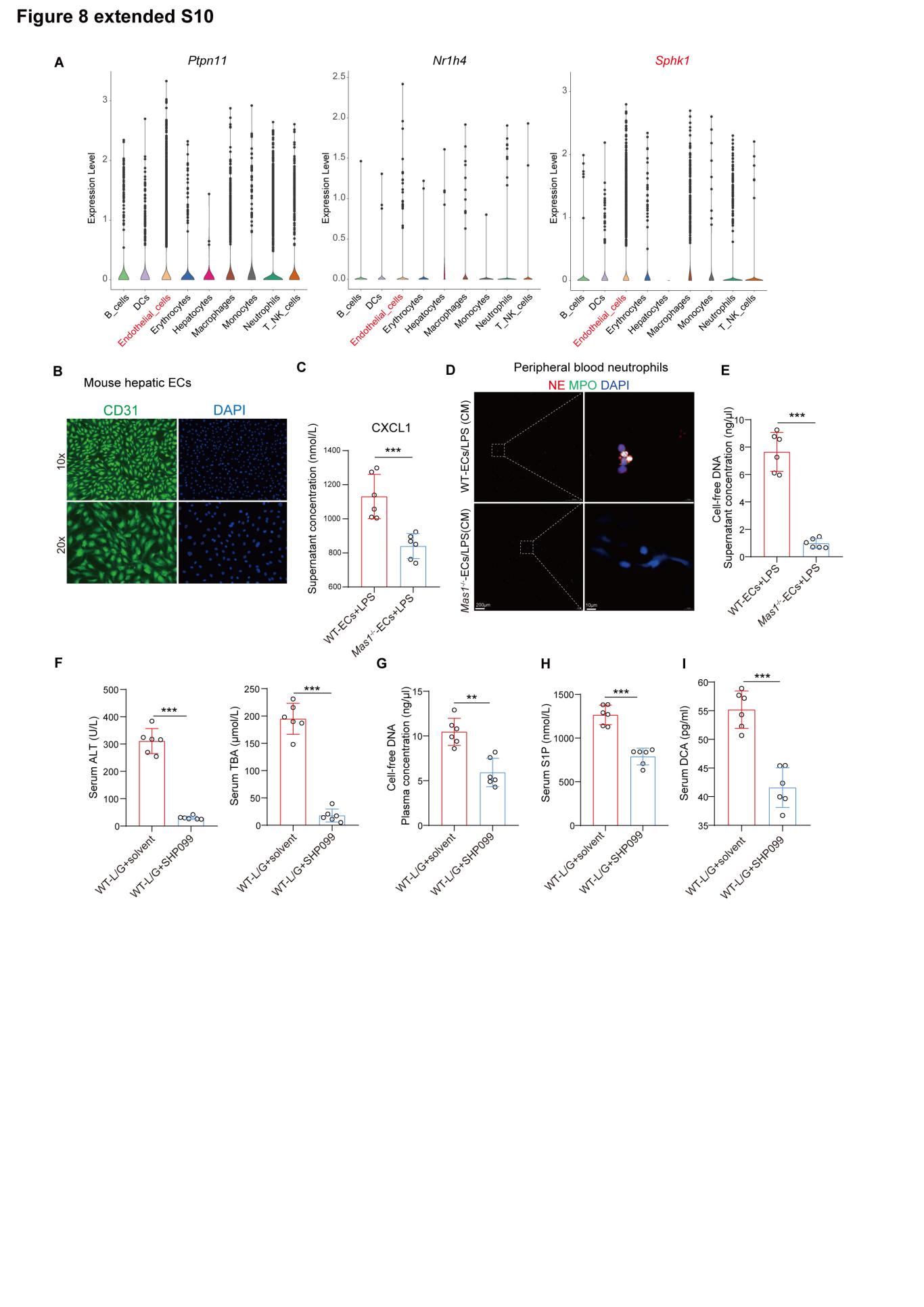
**Figure S10. Related to Figure 8.** Intrahepatic SHP2 functions downstream of Mas to regulate the FXR-S1P-NETs axis during L/G challenge. A) Violin boxplot illustrates the expression levels of *Ptpn11*, *Nr1h4* and *Sphk1* across different cell types. B) Staining of CD31 in primary hepatic ECs. C) The levels of CXCL1 in the supernatant of LPS-stimulated ECs from WT and *Mas1*^-/-^ mice (two-sided Student’s t-test, p = 8.10 × 10^−4^). D) Representative mIHC staining of NE^+^MPO^+^ neutrophils. Scale bars are shown as indicated. E) The levels of cfDNA in the supernatant of peripheral blood neutrophils (PBNs) (two-sided Student’s t-test, p = 4.19 × 10^−4^). WT mice were treated with SHP099 (SHP2 inhibitor) or solvent control before L/G challenge (n = 6 per group, F-I). F) Serum levels of ALT and TBA (two-sided Student’s t-test, p = 5.62 × 10^−4^ and p = 8.37 × 10^−4^ from left to right). G) Plasma levels of cell-free DNA (two-sided Student’s t-test, p = 3.87 × 10^−3^). H) Serum levels of S1P (two-sided Student’s t-test, p = 5.98 × 10^−4^). I) Serum levels of DCA (two-sided Student’s t-test, p = 6.43 × 10^−4^). Data are presented as mean ± SD (**p < 0.01, ***p < 0.001.).


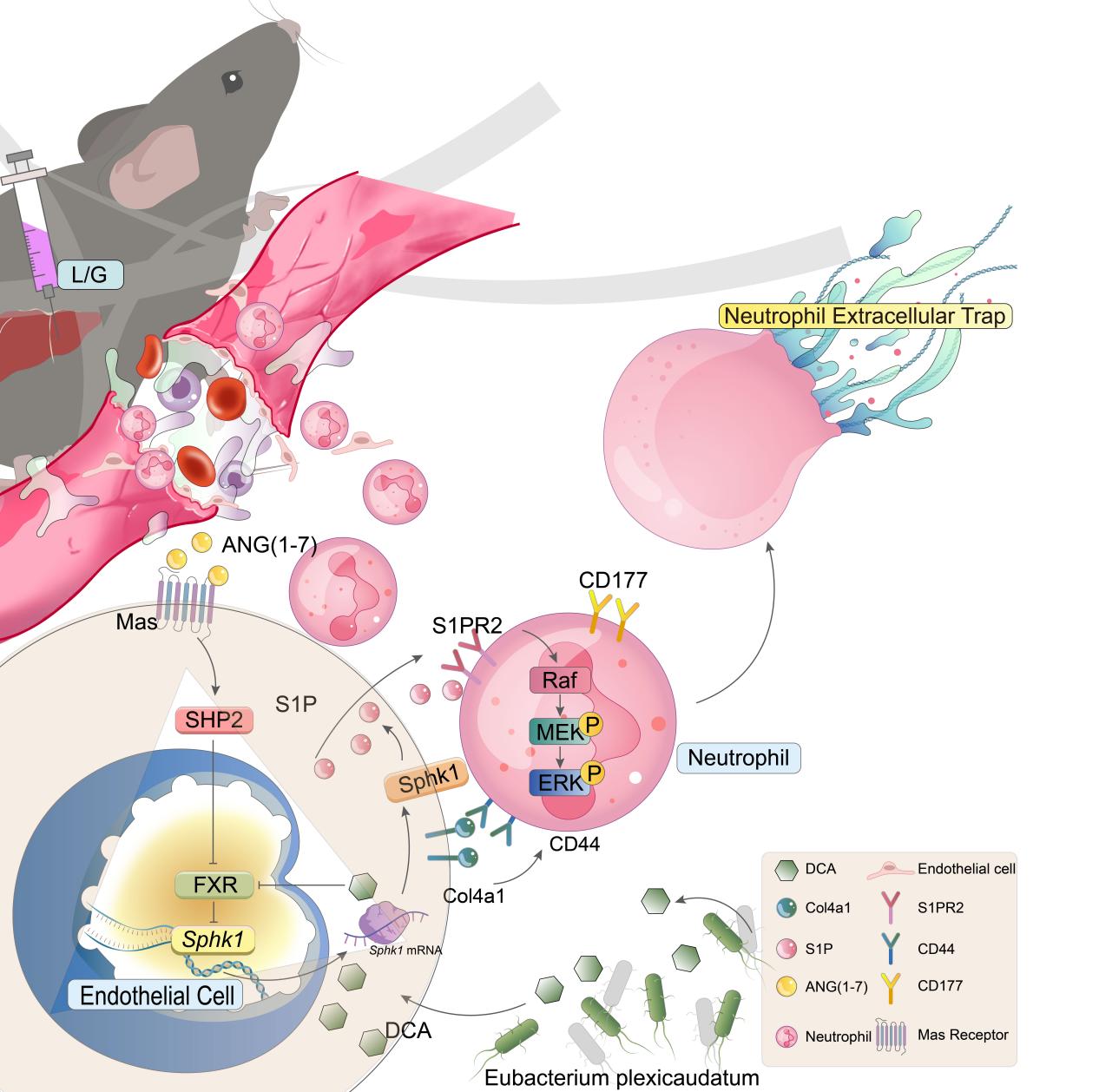


**Figure S11.** Schematic of Mas signaling facilitated S1P-dependent NETs formation in L/G-challenged mice.

In mice exposed to L/G, Mas signaling increased SHP2 in hepatic Col4a1^+^ endothelial cells, inhibiting FXR signaling. FXR inhibition can also occur due to enterogenic DCA. This suppression triggers the S1P-induced Raf/MEK/ERK signaling in Cldn1^+^CD177^+^ neutrophils, leading to neutrophil extracellular trap formation and worsening liver inflammation. Additionally, Mas signaling promotes interactions between endothelial cells and neutrophils through Col4a1-Cd44.
